# Evaluation of heterologous expression in *Pichia pastoris* of Pine Weevil TRPA1 by GFP and flow cytometry

**DOI:** 10.1186/s12934-024-02382-5

**Published:** 2024-04-12

**Authors:** Balder Werin, Wilhelm Hansson Wennersten, Robin Olsson, Oliwia Kołodziejczyk, Martin N. Andersson, Magnus Carlquist, Urban Johanson

**Affiliations:** 1 Center for Molecular Protein Science, Department of Chemistry, Lund University, Lund, SE-221 00 Sweden; 2 Department of Biology, Lund University, Lund, SE-223 62 Sweden; 3 Division of Applied Microbiology, Department of Chemistry, Lund University, Lund, SE-221 00 Sweden

**Keywords:** Flow cytometry, Green Fluorescent Protein, *Hylobius abietis*, TRPA1, Membrane protein, Viability, Ankyrin repeat domain

## Abstract

**Background:**

The wasabi receptor, also known as the Transient Receptor Potential Ankyrin 1 (TRPA1) ion channel, is a potential target for development of repellents for insects, like the pine weevil (*Hylobius abietis*) feeding on conifer seedlings and causing damage in forestry. Heterologous expression of TRPA1 from pine weevil in the yeast *Pichia pastoris* can potentially provide protein for structural and functional studies. Here we take advantage of the Green Fluorescent Protein (GFP) tag to examine the various steps of heterologous expression, to get more insight in clone selection, expression and isolation of the intact purified protein.

**Results:**

The sequence of HaTRPA1 is reported and GFP-tagged constructs were made of the full-length protein and a truncated version (Δ1-708 HaTRPA1), lacking the N-terminal ankyrin repeat domain. Clones were screened on GFP expression plates, induced in small liquid cultures and in fed-batch cultures, and evaluated by flow cytometry and fluorescence microscopy. The screening on plates successfully identifies low-expression clones, but fails to predict the ranking of the best performing clones in small-scale liquid cultures. The two constructs differ in their cellular localization. Δ1-708 HaTRPA1 is found in a ring at the perimeter of cell, whereas HaTRPA1 is forming highly fluorescent speckles in interior regions of the cell. The pattern is consistent in different clones of the same construct and persists in fed-batch culture. The expression of Δ1-708 HaTRPA1 decreases the viability more than HaTRPA1, and in fed-batch culture it is clear that intact cells first express Δ1-708 HaTRPA1 and then become damaged. Purifications show that both constructs suffer from degradation of the expressed protein, but especially the HaTRPA1 construct.

**Conclusions:**

The GFP tag makes it possible to follow expression by flow cytometry and fluorescence microscopy. Analyses of localization, cell viability and expression show that the former two parameters are specific for each of the two evaluated constructs, whereas the relative expression of the constructs varies with the cultivation method. High expression is not all that matters, so taking damaged cells into account, something that may be linked to protein degradation, is important when picking the most suitable construct, clone, and expression scheme.

**Supplementary Information:**

The online version contains supplementary material available at 10.1186/s12934-024-02382-5.

## Background

Membrane proteins are well known for being difficult to express and purify [1], and the process is not made easier by the fact that the quality of the expression often cannot be judged until the protein has been fully purified. There are methods that can help in evaluating the expression process, but they are often not employed in concert to complement each other’s shortcomings. The need for a more holistic approach emerged for us when working with the Transient Receptor Potential Ankyrin 1 ion channel from pine weevil (*Hylobius abietis*; HaTRPA1). The pine weevil is an insect responsible for severe damage to conifer trees [2], and understanding of its putative sensors for detrimental plant-derived chemicals could help develop effective repellents. When we expressed HaTRPA1 in *Pichia pastoris* (*Komagataella phaffii*), we realized that our toolbox had to be expanded to address the low protein yields that we experienced.

TRPA1 is a noxious pain receptor that was first discovered by Jordt et al. [3], and is known in humans to be activated by pungent chemicals, such as allyl isothiocyanate (AITC) of mustard oil and wasabi [3, 4]. The receptor has caught interest for its promiscuous activation, including temperature [5], and its involvement in pain and inflammation [6]. So far, the TRPA1 structures of human and *Drosophila melanogaster* have been solved [7, 8], but the temperature activation is still much debated [5]. The N-terminal Ankyrin Repeat Domain (ARD) is suggested to be involved in both chemical and thermal sensing [6], but TRPA1 from human [9], and *Anopheles gambiae* [10], have been shown to respond on agonists and temperature without it. With this in mind, this study includes a truncated construct of HaTRPA1 without the ARD, with the belief that it will be easier to express and purify [9].

Another valuable addition to the constructs is GFP (Green Fluorescent Protein). GFP was initially discovered by Shimomura et al. [11], and has since become a highly appreciated tool in many areas, among them heterologous protein expression [12, 13]. By creating fusion proteins with GFP, the protein of interest can be followed not only throughout the purification processes, but even inside the cells of the expression host [12–14]. A good example of this is the possibility of easily screening for expression levels in colonies on plates [15]. To get more detailed information, flow cytometry is a method that has been used on *P. pastoris* to determine expression levels in individual cells, but also to estimate the number of intact cells in a cell culture [16].

GFP has been used by Brooks et al. [17] to do expression screening in *P. pastoris*, making it possible to screen hundreds of clones for so-called “jack-pot clones”, i.e. clones with very high expression [17, 18]. Inspired by their work, we complemented their method with fluorescence microscopy and flow cytometry. The fluorescence microscopy allows us to investigate variations in localization in *P. pastoris* between the two constructs with and without ARD. Flow cytometry, on the other hand, gives us information about expression on the individual cell level, as well as on the population level, and gives us valuable information on how the viability of the cells is affected by the expression. Combining these methods, we get a powerful toolbox, that can help us get a lot of information from a limited set of expression experiments, and reveals more dimensions of heterologous protein expression than we could see before. This will also facilitate future optimizations of protocols for expression and purification of difficult membrane proteins, like HaTRPA1.

## Methods

### Cloning and the constructs

The coding sequence of HaTRPA1 was deduced from RNA-seq of mRNA isolated from the antenna of 20 *H. abietis* females, collected in 2016 at Balungstrand’s sawmill in Enviken, close to Falun in mid-Sweden, and kindly provided by Prof. Göran Nordlander (Swedish Univ. Agricultural Sciences). Details on RNA extraction, Illumina RNA sequencing, and assembly have been described previously [19]. The sequence was ordered codon optimized for *P. pastoris*, synthesized and delivered as a pPICZB derivative with an encoded TEV cleavable N-terminal 10× His-tag (HaTRPA1_HaTRPA1 opt_pPICZB; GenScript, Netherlands). A truncated version (HaTRPA1_Δ1-708 HaTRPA1 opt_pPICZB) lacking the N-terminal Ankyrin repeat domain was created by a 2124 bp deletion between two internal NcoI sites.

The coding sequences for full length HaTRPA1 (HaTRPA1) and the truncated version (Δ1-708 HaTRPA1) were amplified by PCR from the plasmids HaTRPA1_HaTRPA1 opt_pPICZB and HaTRPA1_Δ1-708 HaTRPA1 opt_pPICZB respectively, using Phusion High–Fidelity DNA Polymerase (ThermoFisher Scientific), two different forward primers including an identical yeast initiation site (upper case) and an EcoRI site (underlined) HaTRPA1: gcggaattcAAAAATGTCTaactcctttagaagccttgtttca, Δ1-708 HaTRPA1: gcggaattcAAAAATGTCTgctcacggcagagttgaattg and a common reversed primer (cgcctcgagtgttgagtttgccctgtttgag) adding a XhoI site (underlined). The gel purified PCR products were ligated into the corresponding sites of pPICZA_eGFP, a plasmid encoding an enhanced version of GFP, which was kindly provided by Prof. M. Joanne Lemieux (Univ. of Alberta, Canada) [17]. This subcloning omitted the original N-terminal His-tag and TEV site and instead created an in-frame extension coding for a TEV-cleavable GFP-tag with an 8×His-tag at the C-terminus. Both GFP-tagged constructs were transformed into XL1-Blue and plasmids from clones selected on low salt LB plates supplemented with 25 µg/mL Zeocin were verified by sequencing. The purified plasmids were linearized by SacI and used to transform *P. pastoris* (*K. phaffii*) X-33 cells by electroporation (EasySelect™ Pichia Expression Kit manual, ThermoFisher Scientific). Transformants were selected on YPDS plates supplemented with 100 µg/mL Zeocin according to the EasySelect™ Pichia Expression Kit manual (ThermoFisher Scientific).

### On-plate GFP screening

In an initial screening 25 transformants of each construct were streaked on BMMY agar plates. Two clones from each construct with a high and low fluorescence were chosen as positive and negative controls respectively, and used as references in subsequent screenings of additional 96 Δ1-708 HaTRPA1 and 48 HaTRPA1 clones. The plates were incubated for 48 h at 30 °C, with 100 µL methanol in the lid, to induce protein expression as previously described [18]. The fluorescence was captured using a Gel imaging system (Syngene, Cambridge, UK), and evaluated by visual comparison.

### Small scale expression

The best clones identified, as well as an intermediate expressing clone from the induction plates were selected for further comparisons. In total 10 different clones from the on-plate induction screening were restreaked on fresh YPD plates, along with one Δ1-708 HaTRPA1 clone without the GFP tag, and the untransformed X-33 strain. We then inoculated and grew the clones in 50 mL tubes with 5 mL BMGY medium each, at 200 rpm and 30 °C for 24 h. Cells corresponding to 5 mL at OD_600_ = 1 were spun down at 845 rcf for 5 min and resuspended in 5 mL BMMY medium to start the induction, and incubated further at 30 °C. 24 and 43 h after the induction, the methanol was replenished by adding 25 µL of 100% methanol to each tube. The cells were harvested 45 h after induction. The cells were spun down and diluted in PBS (137 mM NaCl, 2.7 mM KCl, 10 mM Na_2_HPO_4_, 2 mM KH_2_PO_4_, pH 7.5) to OD_600_ = 0.1 for flowcytometry studies, and OD_600_ = 20 for fluorescence microscopy studies.

### Fed-batch cultivation and induction

The large-scale fermentation was done at 30 °C in a 3 L bench top bioreactor (Belach bioteknik, Sweden). 1.5 L basal salt medium (7 mM CaSO_4_, 0.1 M K_2_SO_4_, 60 mM MgSO_4_ × 7 H_2_O, 74 mM KOH, 4% glycerol, 2.3% H_3_PO_4_) supplemented with PTM (24 mM CuSO_4_, 0.5 mM NaI, 18 mM MnSO_4_ × H_2_O, 0.8 mM Na_2_MoO_4_ × 2 H_2_O, 0.3 mM H_3_BO_4_, 2 mM CoCl_2_, 150 mM ZnCl_2_, 234 mM FeSO_4_ × 7 H_2_O, 0.8 mM Biotin, 0.4% H_2_SO_4_) was inoculated with 100 mL pre-culture grown overnight in YPD [9, 10, 20]. The medium contained 60 mL glycerol, and when it had been consumed, a feed with 50% glycerol supplemented with PTM was started, at a rate keeping the dissolved oxygen level above 20%. When 200 ml glycerol had been added and consumed, the feed was switched to 100% methanol supplemented with PTM to start the induction. The methanol feed was maintained for the rest of the fermentation, for a total of 44.67 h. The culture was harvested by centrifugation in a JLA 8.1000 rotor at 6,000 rpm (6,900 rcf) for 30 min and stored at -80 °C.

### Flow cytometry analysis

Cells from the small-scale expression or the fed-batch cultivation were collected, stored on ice, and measured within 8 h. 6 µL PI (Propidium iodide) stock solution (1.5 mM) was added to 500 µL cell sample, and incubated dark for at least 15 min before measurements. The measurements from the small-scale fermentation were performed on a MACSQuant VYB (Miltenyi Biotec, Bergisch Gladbach, Germany) with three excitation lasers at 405 nm, 488 nm and 561 nm and eight emission channels. 20,000 events were collected per sample at a flow rate of 25 µL/min, and the trigger was set to 1.0 in FSC-A. The measurements from the fed-batch cultivations were performed on a BD Accuri C6 Plus Flow cytometer (BD biosciences, New Jersey, USA). Measurements were collected on 10 µL sample and the trigger was set to 80,000 in FSC-H. For all measurements, detection filters 510/15 nm (FL1) and 610/20 nm (FL3) were used. The data was analyzed with FlowJo v10.9.0 (BD Life Sciences). First, a gate was made in FSC-A vs. SSC-A, to include only the Pichia cells (Figure S1). For the small-scale induction, we then gated based on PI signal (Figure S2).

### Statistical analysis

**Fig. 1:**
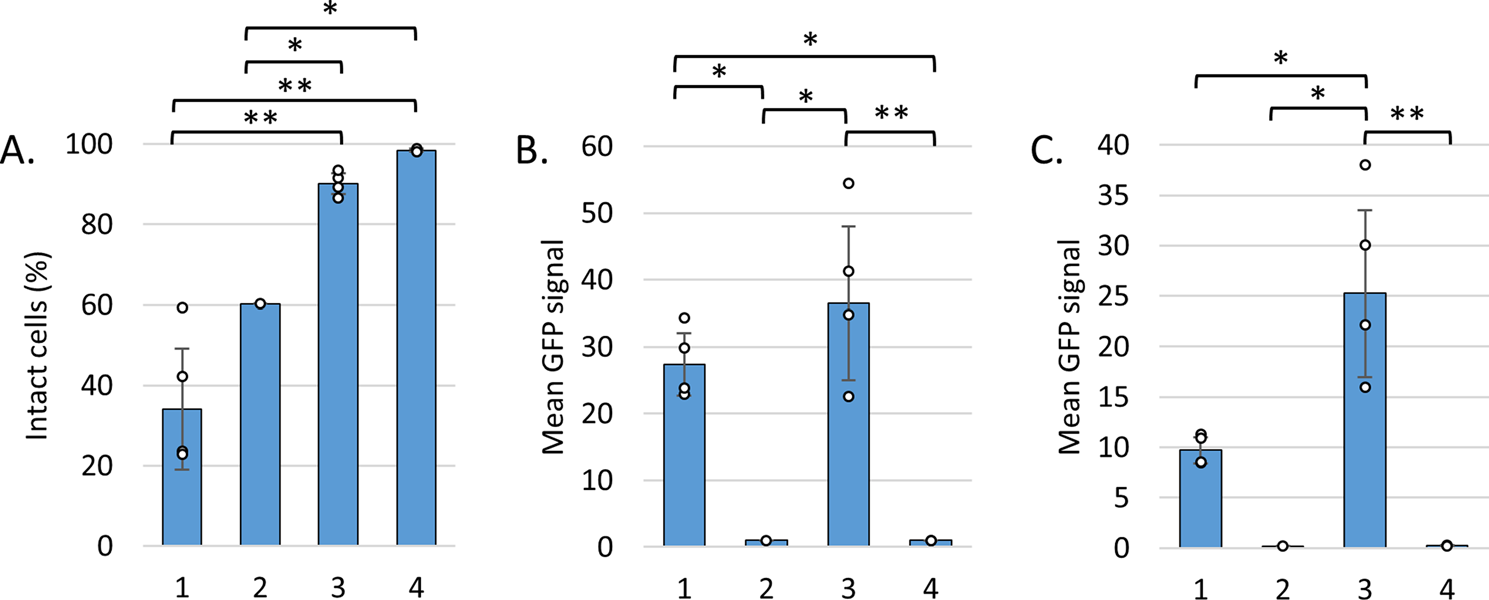
Flow cytometric analysis of small-scale induction. (**A**) Cell viability. The relative number of cells with FL3-H (PI) values lower than the gate. The Δ1-708 HaTRPA1 cell samples have less PI negative cells, indicating a lower viability. (**B, C**) The mean GFP signal of the different types of constructs, only gated on FSC-A and SSC-A (**B**), and gated on FL3-H as well (**C**). Both constructs have higher GFP signals than the negative controls (**B**), but the HaTRPA1 cell samples have the highest GFP signal, considering only viable cells (**C**). The brackets indicate differences with statistical significance levels * corresponding to *p* < 0.05, and ** corresponding to *p* < 0.005 from a single factor ANOVA with Tukey-Kramer post hoc analysis. (1) All Δ1-708 HaTRPA1 clones except BW30 (corresponding clone without GFP tag) and 2.1.12, (2) Δ1-708 HaTRPA1 BW30, (3) all HaTRPA1 clones except 2.3.28, (4) Δ1-708 2.1.12 and HaTRPA1 2.3.28 (the low fluorescing negative reference clones)

In order to determine the significance of the data from the flow cytometry in Fig. 1, a built-in single factor ANOVA was performed in Excel (Microsoft) with α = 0.05, and a Tukey-Kramer post hoc analysis. The significance levels are presented with * corresponding to *p* < 0.05 and ** corresponding to *p* < 0.005.

### Fluorescence microscopy

PBS with 1% agarose was used to make a pad on a microscope slide. 10 µL sample with an OD_600_ of 20 was placed on the pad and covered with a cover slip. Microscope images were collected using separate channels for phase contrast and fluorescence in a Zeiss Axio Imager.Z1 microscope equipped with X-Cite 120 Illumination (EXFO Photonic Solutions Inc.) and a 9100-02 EM-CCD camera (Hamamatsu Photonics). Data was handled with the Zen microscopy software and images analyzed using Fiji (ImageJ) [21].

### Membrane preparation

100 g of cells were lysed in a Bead beater (BioSpec Products) with breaking buffer (50 mM NaH_2_PO_4_, 1 mM EDTA, 5% glycerol, 1 mM PMSF, pH 7.4), and the cell debris was pelleted at 9,500 rpm (7,386 rcf) for 30 min in a JA 25.50 rotor. The supernatant was centrifuged at 45,000 rpm (158,024 rcf) for 1 h in a 45 Ti rotor, to pellet the membrane. The crude membrane was homogenized, washed in buffer A (20 mM HEPES, 0.5 M NaCl, 10% glycerol, 2 mM β-mercaptoethanol, 1 mM PMSF, pH 7.8), and then centrifuged again at 45,000 rpm (158,024 rcf) for 1.5 h in a 45 Ti rotor. The washed membrane pellet was resuspended in 2 mL buffer A per g membrane, homogenized, snap-frozen in liquid nitrogen and stored at -80 °C.

### Solubilization and purification

Washed membrane was mixed with 70 mL buffer A with 1% Fos-choline 14, and EDTA-free protease inhibitor cocktail tablets (cOmplete, Roche) added according to the manufacturer’s instructions, for 3 h at room temperature to solubilize the protein. Unsolubilized protein was pelleted at 30,000 rpm (70,233 rcf) for 30 min in a 45 Ti rotor and the supernatant was incubated with Ni-NTA agarose overnight on a slowly rotating axis at 4 °C. The agarose was packed in a gravity flow column and washed with buffer B (20 mM HEPES, 0.3 M NaCl, 10% glycerol, 2 mM β-mercaptoethanol, 1 mM PMSF, pH 7.8) containing 30 mM imidazole and 3×CMC Fos-choline 14 (0.0138%), and the protein was eluted with buffer B with 300 mM imidazole and 3×CMC Fos-choline 14 (0.0138%). The sample was concentrated and run on a Superose 6 increase 10/300 (GL Healthcare, Little Chalfont UK) column, with elution buffer TBS (20 mM Tris, 150 mM NaCl, pH 7.5) with 3×CMC Fos-choline 14 (0.0138%).

### SDS-PAGE

Samples for SDS-PAGE were prepared by mixing 22 µL sample with 11 µL solubilization buffer 3 × (0.375 M Tris, 200 mM SDS, 30% glycerol, pH 6.8, 0.1% bromophenol blue, 7.5 mM β-mercaptoethanol), and incubating at room temperature for 30 min. The gels were run with running buffer (25 mM Tris, 0.19 mM glycine, 3.5 mM SDS, pH 8.3) on 10% precast polyacrylamide gel (Mini-PROTEAN TGX, Bio-Rad) at 200 V. In-gel fluorescence was recorded on a ChemiDoc Imaging System (BioRad, Hercules California USA), after which Coomassie staining was carried out.

### Microtiter plate quantification

A sample from the solubilized protein, and a sample from the pellet with unsolubilized protein resuspended in 500 µL buffer A, of each construct was diluted 1:20 in TBS with 3×CMC Fos-choline 14. Fluorescence was measured on the samples in a 96-well plate with black walls and clear bottom on a CLARIOstar microplate reader (BMG Labtech, Ortenberg, Germany) with the excitation of 470 − 15 and emission of 515 − 20.

## Results

### Construction of GFP-tagged HaTRPA1 expression clones of *P. pastoris*

**Fig. 2:**
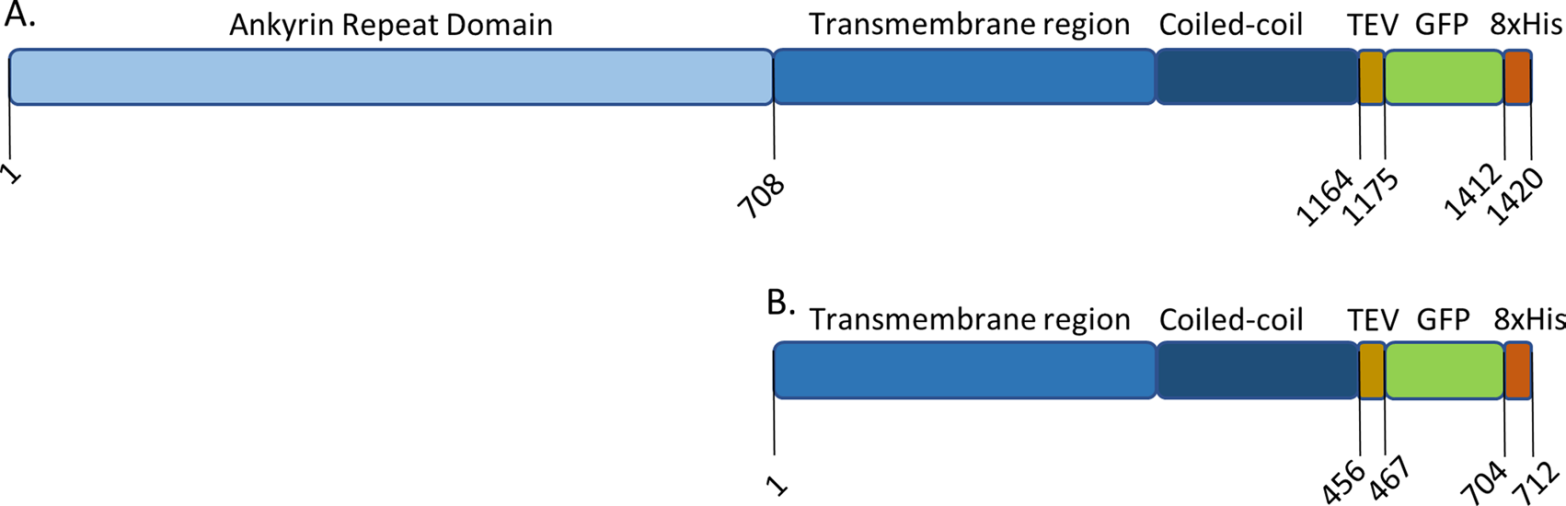
Schematic representation of the constructs. Illustration showing locations of predicted domains and added C-terminal tags in the two constructs used in this study (**A**) HaTRPA1, (**B**) Δ1-708 HaTRPA1. Δ1-708 HaTRPA1, which is about half the size of the full length is lacking the Ankyrin repeat domain (ARD). Sequences of the constructs in FASTA format are provided in supplemental text file

The coding sequence for HaTRPA1 was determined by RNA-seq of the transcriptome of the female antenna. To facilitate the expression in *P. pastoris* a codon optimized version was synthesized and in addition a truncated version was made (Δ1-708 HaTRPA1), lacking the N-terminal Ankyrin repeat domain. A corresponding truncation had previously been shown to increase the expression levels of TRPA1 orthologs from man and malaria mosquito in *P. pastoris*, while retaining its ability to respond on activating stimuli [9, 10]. The coding sequence of each construct (full-length HaTRPA1 and Δ1-708 HaTRPA1) was subcloned behind the strong methanol inducible AOX1 promoter pPICZA-eGFP to obtain fusions encoding a cleavable GFP- and His-tag at the C-terminus (Fig. 2, Sequences in Supplemental text). These two derivatives of pPICZA-eGFP were then introduced into *P. pastoris* by electroporation. For Δ1-708 HaTRPA1 about 600 colony forming units (CFU) were obtained whereas the result for the full-length construct was only about a third of that.

### GFP screening on plates

**Fig. 3:**
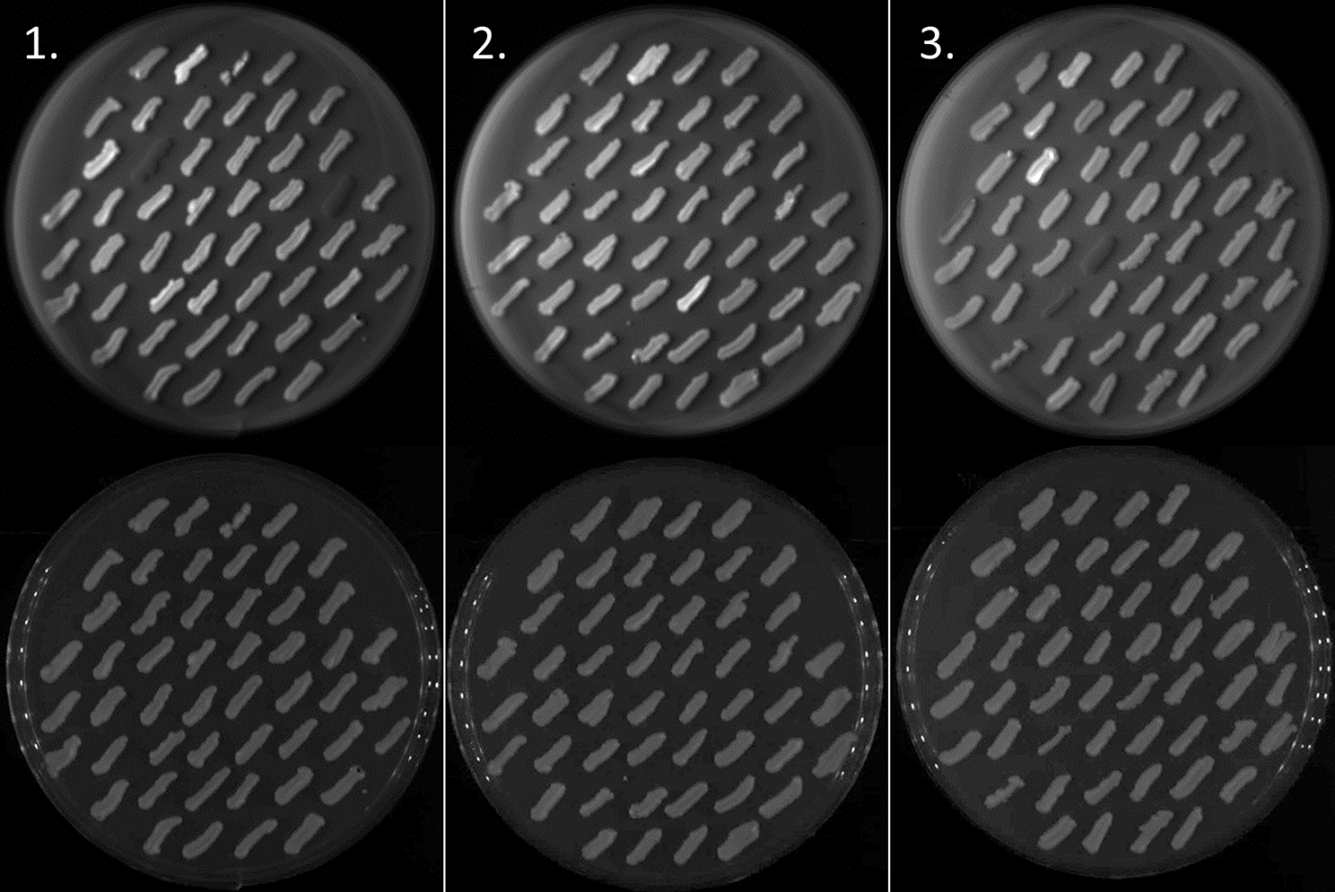
Screening of GFP expression on plates. Top row, fluorescence imaging. Bottom row, white light imaging of the same plates. The left (plate 1) and middle (plate 2) column contain 48 clones each of Δ1-708 HaTRPA1, and the right (plate 3) column contains 48 clones of HaTRPA1. On each plate, the first clone is a low GFP control, and the second clone is a high GPF control from the first screen. Plate 1 clone 11, plate 2 clone 37 has similar intensity to the high control, and plate 3 clone 12 has higher intensity than the high control. Plate 1 clone 12 and 23, and plate 3 clone 28 and 35 have virtually no expression. On each plate clones are numbered starting from the top left

To identify individual clones having high expression levels, so-called *jack-pot clones*, the transformants were streaked onto BMMY plates and induced with extra methanol added in the lid of the reversed plates. This was iterated to screen more than 50 and 100 transformants for HaTRPA1 and Δ1-708 HaTRPA1, respectively. The GFP-fluorescence of the final screening plates is shown in Fig. 3, where two Δ1-708 HaTRPA1 clones of high intensity and one HaTRPA1 clone of very high intensity are identified. For the small-scale induction in liquid media, four clones each from HaTRPA1 and Δ1-708 HaTRPA1 were picked, along with a weakly fluorescing representative of each construct, and a Δ1-708 HaTRPA1 clone without GFP-tag isolated in previous work. The clones selected are described in Table 1. Note that HaTRPA1 2.3.12 represents the highest fluorescing clone on the plate, whereas Δ1-708 HaTRPA1 2.1.35 represents average fluorescence. To investigate any heterogeneity in expression among cells presumably originating from the same transformed cell, two and three CFUs from restreaks of Δ1-708 HaTRPA1 1.2 and HaTRPA1 1.2, respectively, were included and individually compared.

**Table 1:** Clones in liquid small-scale induction. Descriptions of all clones included in the small-scale induction, and explanations of their origins

Clone	Description
Δ1-708 BW30	Construct corresponding to Δ1-708 HaTRPA1 without GFP
Δ1-708 2.1.12	Clone 12 from plate 1 (low fluorescence)
Δ1-708 2.1.35	Clone 35 from plate 1 (average signal)
Δ1-708 1.2.1	Restreak 1 of clone 1.2^b^
Δ1-708 1.2.2^a^	Restreak 2 of clone 1.2^b^
Δ1-708 2.2.37	Clone 37 from plate 2 (similar to high control)
HaTRPA1 2.3.28	Clone 28 from plate 3 (low fluorescence)
HaTRPA1 2.3.12	Clone 12 from plate 3 (higher than high control)
HaTRPA1 1.2.1^a^	Restreak 1 of clone 1.2^b^
HaTRPA1 1.2.2	Restreak 2 of clone 1.2^b^
HaTRPA1 1.2.3	Restreak 3 of clone 1.2^b^

^a^Used for fed-batch expression

^b^Isolated in the first screening round

### Small-scale induction

To further analyze the clones picked in the screening, flow cytometry was used to measure GFP fluorescence and the viability by propidium iodide (PI) staining of the cells.

The percentage of intact cells in the negative reference clones (Δ1-708 HaTRPA1 2.1.12 and HaTRPA1 2.3.28) with very low expression is close to 100% (Fig. 1A). Looking at the clones selected on plates as GFP positive, there is significant lower percentage of intact cells and a large difference between HaTRPA1 (90%) and Δ1-708 HaTRPA1 (37%), whereas a high expression clone corresponding to Δ1-708 HaTRPA1 but lacking the GFP tag has an intermediate viability (60%). Overall the GFP signal is not significantly different for the entire cell populations in the selected high expression clones of HaTRPA1 compared to Δ1-708 HaTRPA1 (Fig. 1B). Looking at individual clones, it is evident that HaTRPA1 2.3.12, which have the strongest fluorescence of the full-length construct on the plate is not the best in the small-scale liquid culture (Table S1). Among Δ1-708 HaTRPA1 clones there appears to be a negative trend for the viability in response to increasing GFP signal (Figure S3). Between the clones isolated as individual CFUs originating from the same transformed cell there is possibly some difference in the expression level, which could indicate a genetic instability, however to establish this, further investigations are required. The average GFP expression of the intact selected Δ1-708 HaTRPA1 cells is only about a third (35%) of average GFP in all the Δ1-708 HaTRPA1 cells, whereas for HaTRPA1 the corresponding number is about 2-fold higher (69%). Thus, the mean GFP fluorescence in the viable cells is 2.5-fold higher in HaTRPA1 compared Δ1-708 HaTRPA1 clones (Fig. 1C).

**Fig. 4:**
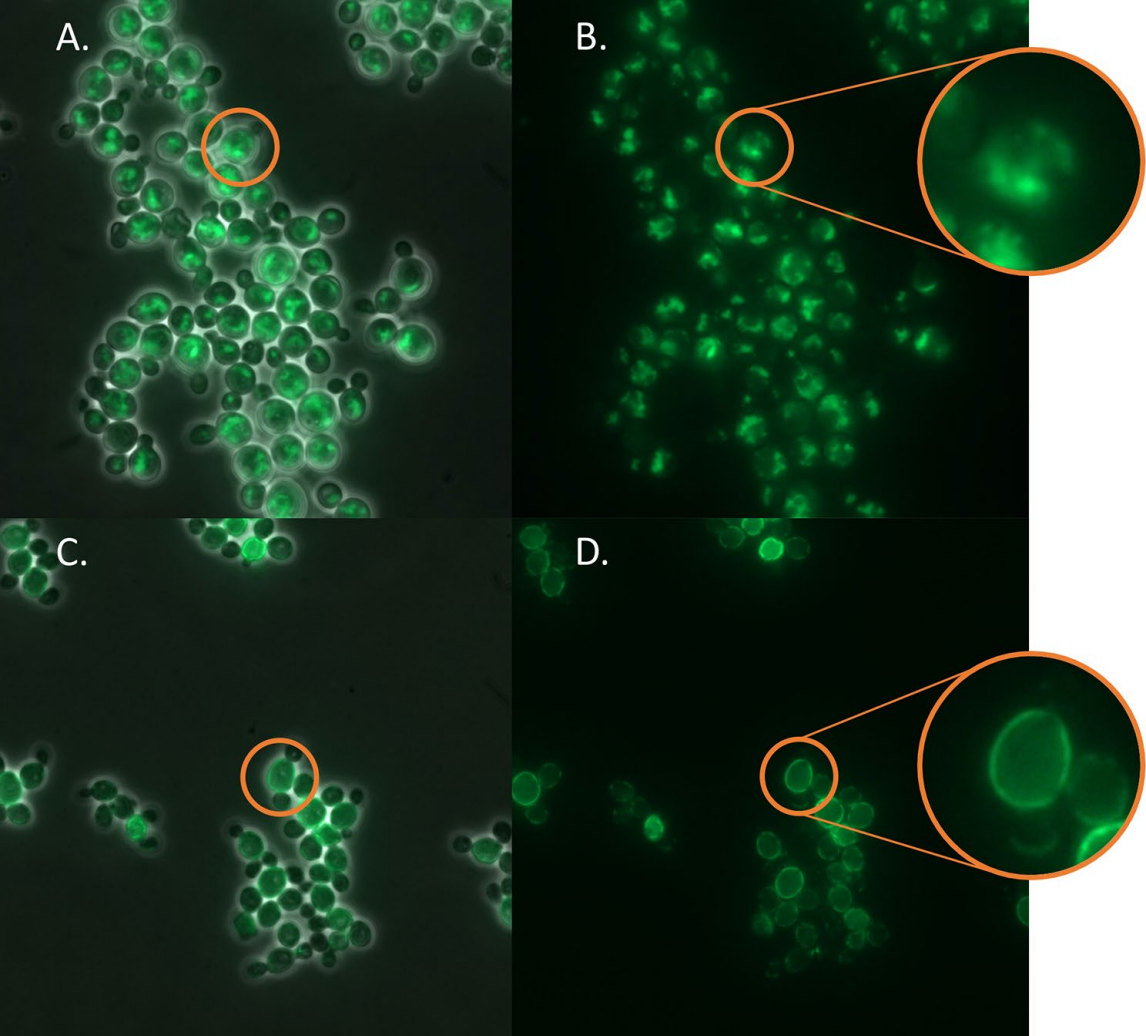
Subcellular localization of GFP signal. Representative images of the GFP signal together with phase contrast signal (**A**, **C**), and GFP signal alone (**B, D**). In HaTRPA1 clones (**A, B**, exemplified by clone HaTRPA1 1.2.1) the fluorescence is localized more to the interior of the cell with some very bright speckles, whereas in Δ1-708 HaTRPA1 clones (**C, D**, exemplified by clone Δ1-708 HaTRPA1 2.2.37) it is localized around the edge of the cell. A single cell of each construct is highlighted and shown in a close up

When analyzing the small-scale cultures with fluorescence microscopy (Fig. 4), the HaTRPA1 cells have fluorescence localized to “speckles” inside the cell, whereas Δ1-708 HaTRPA1 cells have fluorescence arranged in a ring around the edge of the cell. These construct specific patterns were found in all clones with detectable expression levels.

### Fed-batch expression

Based on the small-scale expression, the clone with the consistent highest expression was selected for each of the two constructs and evaluated in a fed-batch bioreactor, withdrawing samples at specified time points to monitor the cells at different stages using flow cytometry.

**Fig. 5:**
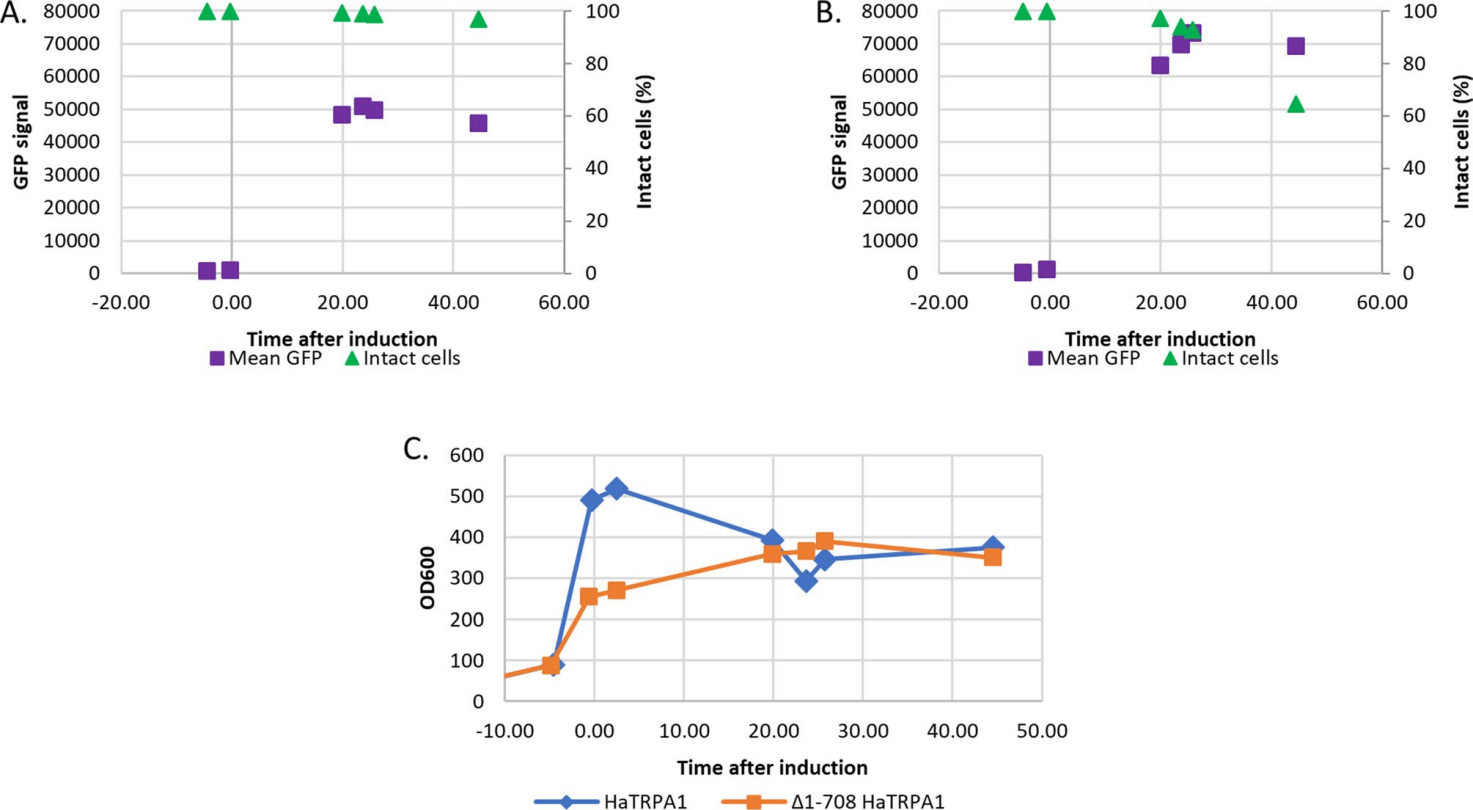
Data from monitoring of the fed-batch cultivations. Graphs indicating mean GFP signal and intact cells (PI negative) in the fermentor cultures of HaTRPA1 (**A**) and Δ1-708 HaTRPA1(**B**). In Δ1-708 HaTRPA1, the number of viable cells is decreasing towards the end of the fermentation. The bottom plot (**C**) shows the OD_600_ of HaTRPA1 and Δ1-708 HaTRPA1 in the fermentor cultures

The percentage of intact cells was monitored throughout the cultivation in order to evaluate potential fitness reduction arising upon induction (Fig. 5). The Δ1-708 HaTRPA1 culture viability features a marked decline towards the end of the fermentation, with the number of intact cells at the endpoint reaching around 64%. For the HaTRPA1 cultivation the reduction in viability is much more modest, retaining 97% intact cells at the end. This reflects the results of the small-scale induction, where HaTRPA1 clones showed higher percentages of intact cells compare to Δ1-708 HaTRPA1 clones, although the fraction of intact cells is smaller in small scale induction. Note that the small-scale measurements were performed at a time corresponding roughly to the endpoint of the large-scale fermentation.

The mean GFP signal of both the HaTRPA1 and Δ1-708 HaTRPA1 cells increases greatly after the induction, but then remains fairly stable throughout the fermentation (Fig. 5). There seems to be no peak in fluorescence, but rather a steady state is reached within 20 h of induction. For HaTRPA1 this is around 50,000 fluorescence units, and around 70,000 fluorescence units for Δ1-708 HaTRPA1. This is the opposite result compared to the small-scale induction where HaTRPA1 performed better than Δ1-708 HaTRPA1, hence the expression levels in small-scale are in this case not foretelling the relative outcome of the fed-batch cultivation.

**Fig. 6:**
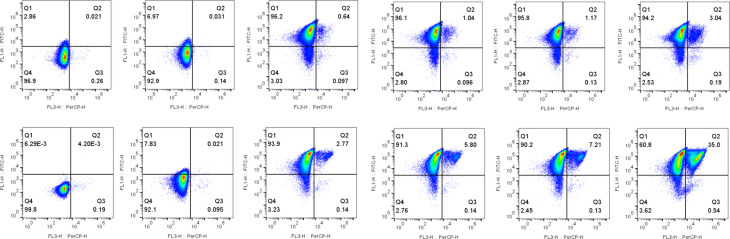
Time series fed-batch flow cytometry bivariate plots. The panels correspond to the time points in Fig. 5A (top row, HaTRPA1) and B (lower row, Δ1-708 HaTRPA1). Note how the cells, most clearly seen in Δ1-708 HaTRPA1, move clockwise from the bottom left quadrant (Q4), through the top left quadrant (Q1) into the top right quadrant (Q2). This means that cells first start to express GFP and HaTRPA1, and then later lose viability. FL1 reports the GFP fluorescence and FL3 the PI staining i.e. damaged cells. The percentage of the total cells is indicated in each quadrant

During the course of induction, the Δ1-708 HaTRPA1 cells start as intact cells with no GFP signal and gradually become intact cells with GFP signal, of which a large fraction eventually ends up as damaged cells with GFP signal (Fig. 6). The first two stages are similar for HaTRPA1, but the fraction of damaged cells with GFP signal is less than a tenth compared to Δ1-708 HaTRPA1.

The OD of the Δ1-708 HaTRPA1 culture seems to be increasing steadily throughout the fermentation, except for the final measurement. The HaTRPA1 culture on the other hand shows a peak in OD, 2.5 h after induction, to later stabilize on similar end levels as Δ1-708 HaTRPA1. This is consistent with the similar weight of the cell pellets of the two constructs (HaTRPA1, 552 g and Δ1-708 HaTRPA1, 539 g).

When looking at fluorescence microscopy, the same subcellular pattern that distinguishes HaTRPA1 from Δ1-708 HaTRPA1, that we saw in small scale, is present in the fermentation as well in the final stages of expression (Figure S4).

### Purification

**Fig. 7:**
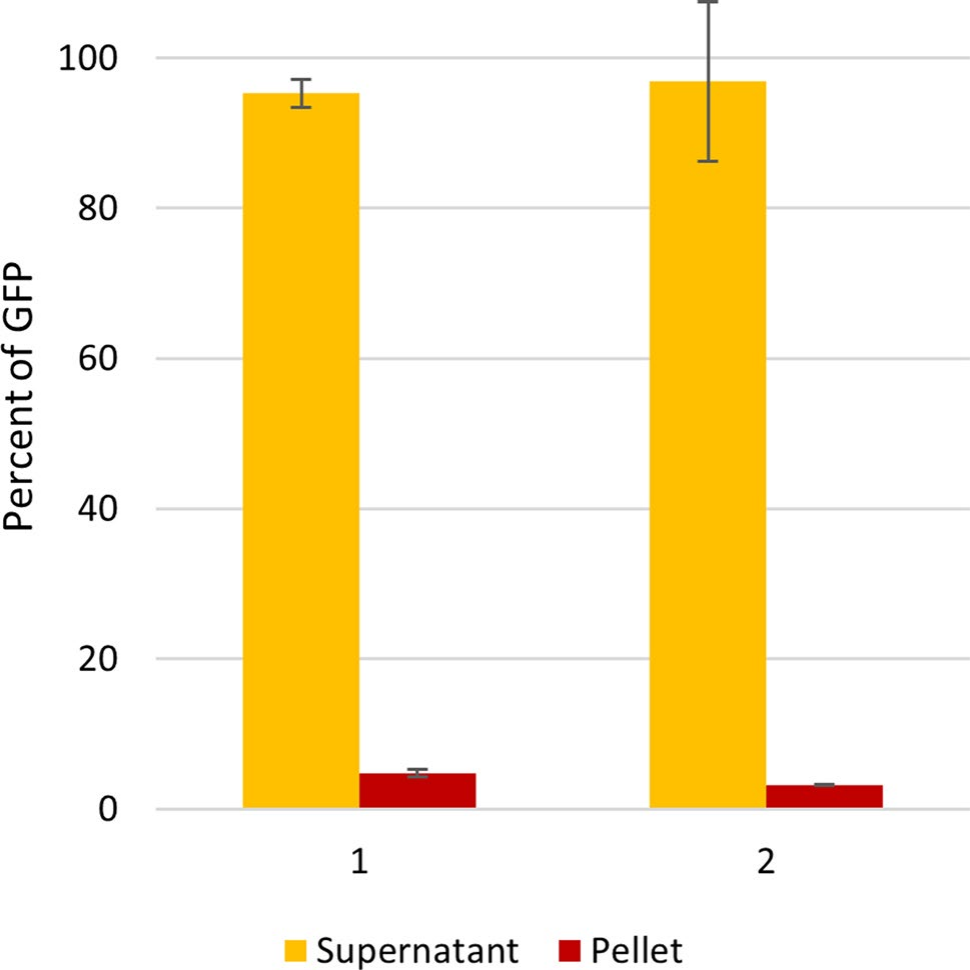
Microtiter plate fluorescence measurements of solubilization samples. Most of the GFP signal is in the supernatant of HaTRPA1 (1) and Δ1-708 HaTRPA1 (2)

To evaluate the quality of the expressed proteins, washed membranes were isolated from the fed-batch cultures. Fos-choline 14 was used to solubilize HaTRPA1 and Δ1-708 HaTRPA1 at room temperature and with added protease inhibitors. For both constructs more than 90% of the GFP-tagged protein was successfully solubilized according to the fluorescence of the different fractions (Fig. 7).

**Fig. 8:**
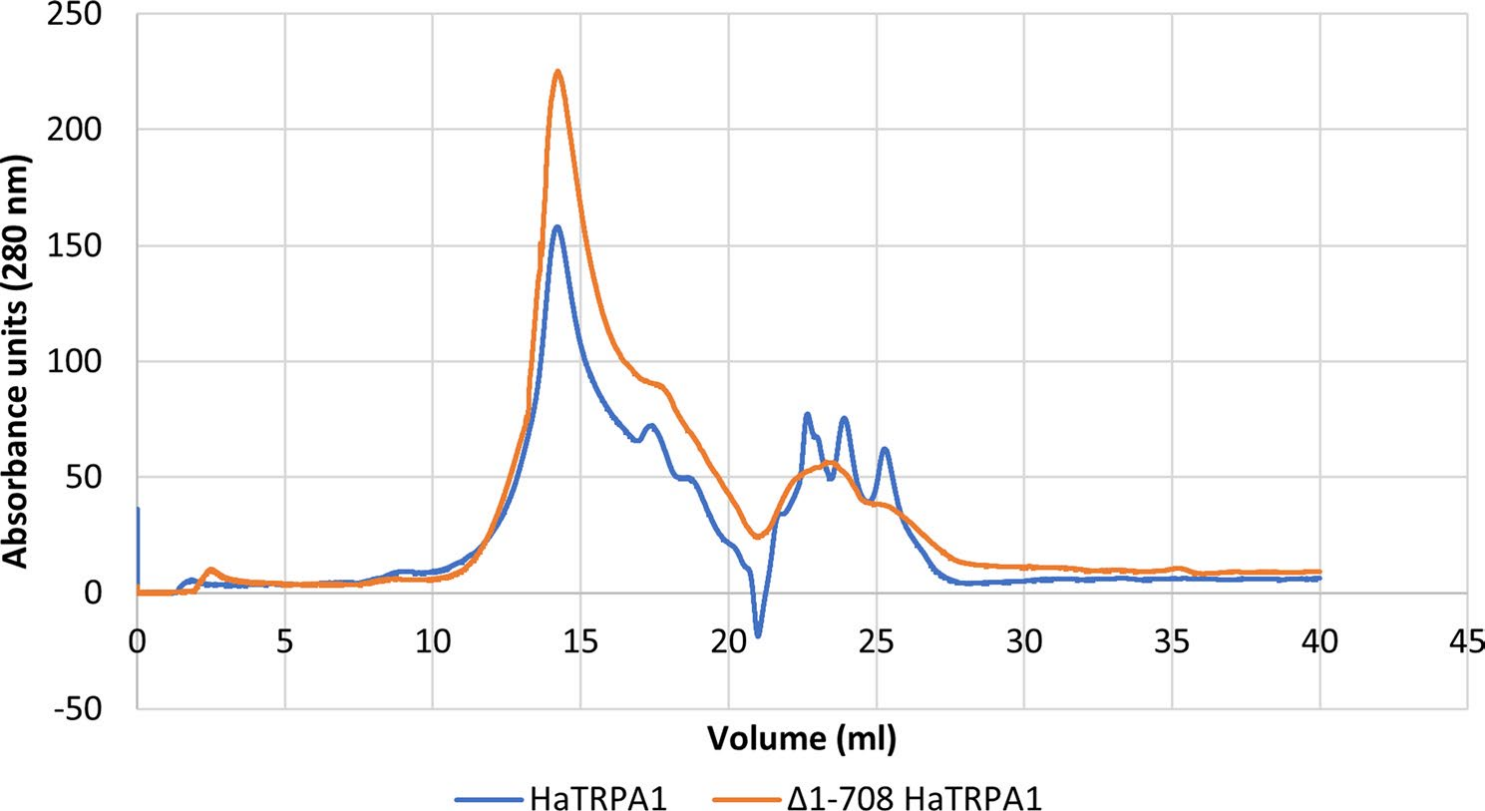
SEC chromatograms. Shows a peak close to the expected elution volume of tetrameric HaTRPA1 without GFP. Other peaks may be from monomers, free GFP etc. or from aggregates. Note that the main peak for both constructs arrives around 14 mL, which implies a similar size

After purification with IMAC, analytical SEC (Size Exclusion Chromatography) was performed on the concentrated eluate. Both HaTRPA1 and Δ1-708 HaTRPA1 give a major elution peak around 14 mL (Fig. 8). The elution volumes are very similar, despite the expected two-fold difference in size of the constructs, and more alike the theoretical elution volume of tetrameric HaTRPA1 without GFP.

**Fig. 9:**
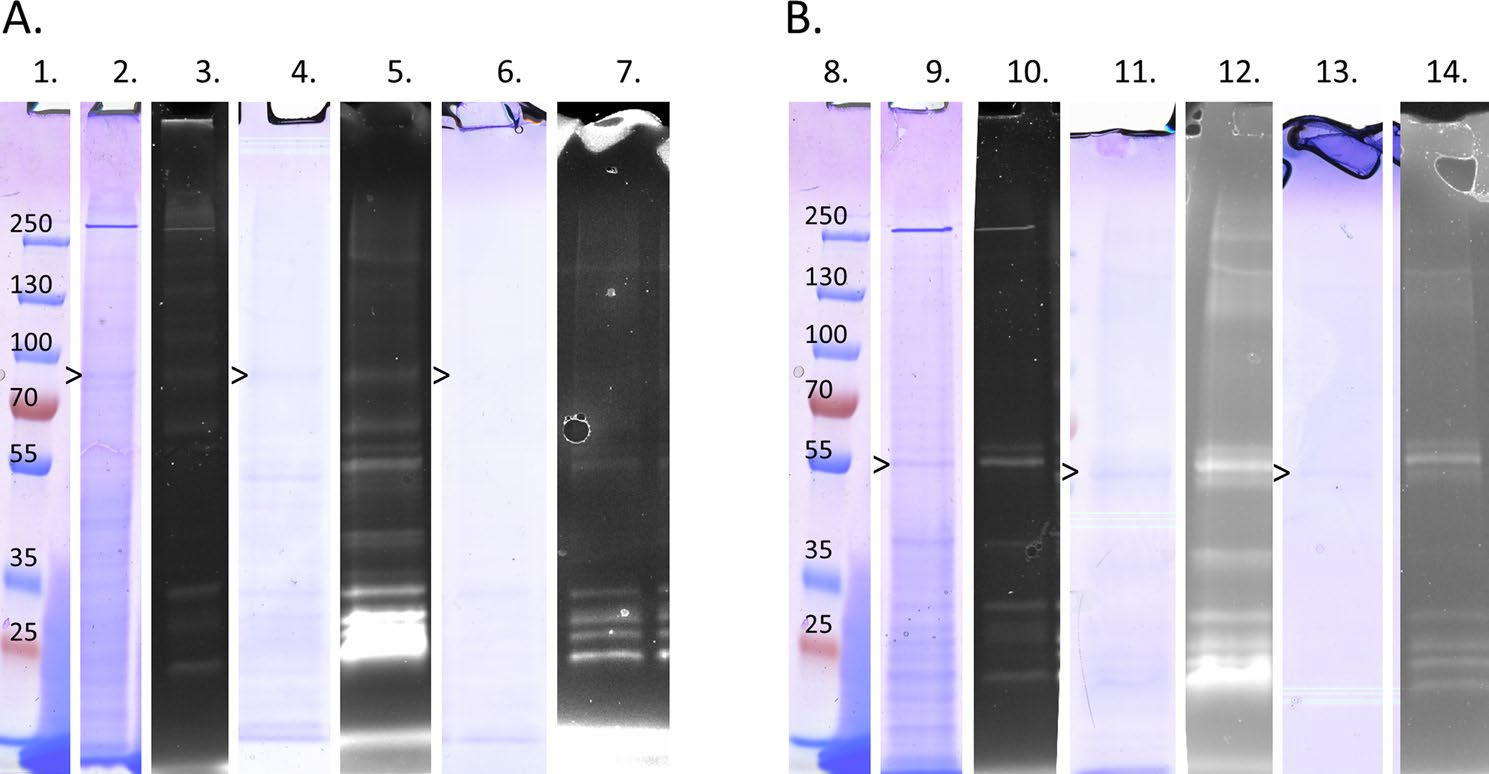
Fluorescent gels and Coomassie stained gels from the purification show what remains in each step of the purification. Both the samples, HaTRPA1 and Δ1-708 HaTRPA1, contain a lot of smaller bands throughout the purification. (**A**) HaTRPA1 does not show any bands that obviously correspond to the correct size of the construct, but after the IMAC there is a band marked by an arrowhead just below 100 kDa, which is far smaller than expected. (**B**) Δ1-708 HaTRPA1 in the meantime, contains a band around 60 kDa throughout the purification, which is somewhat smaller than expected. (1 and 8) PageRuler Plus Prestained Protein Ladder (Thermo Fisher). (2, 3, 9 and 10) Washed membrane, Coomassie stained and fluorescent gel. (4, 5, 11, and 12) Eluate from IMAC, Coomassie stained and fluorescent gel. (6, 7, 13 and 14) Fraction 29 from the SEC, Coomassie stained and fluorescent gel. The full gels are found in Figures S5-S10

Evaluating the purification with SDS-PAGE (Fig. 9), there seem to be a lot of smaller bands through most steps for both HaTRPA1 and Δ1-708 HaTRPA1. HaTRPA1 does not show any bands obviously matching the right size, but in the final step there is a weak band just below 100 kDa, which is far smaller than expected. Δ1-708 HaTRPA1, in the meantime, contains a band around 60 kDa throughout the purification, which is close to, but still a bit smaller than expected.

## Discussion

### Selecting a good clone from the transformation

The method of GFP based screening of transformants is attractive since it lets us select possible candidates from a large set by a signal that is directly linked to the expression of our protein of interest [14, 15, 17, 18]. This method is, of course, not without its limits. The fluorescence intensity would be expected to vary with thickness of the colonies, or any uneven distribution of methanol, and on-plate fluorescence might not directly translate into high yield of purified correctly folded protein using completely different growth conditions.

When comparing the clones chosen from the plate screen with the results from the small-scale expression, it is clear that the clones identified as low fluorescing on the plate indeed have low GFP signal when analyzing the cultures with flow cytometry as well. All of the clones that were fluorescent on plate, also had GFP signal when analyzing the cultures with flow cytometry, but the clones that had the highest fluorescence on plate, were not always the ones with the highest GFP signal in the cultures. This means that we can accurately identify low-expressing clones, but have a harder time distinguishing between the higher expressing clones. Nevertheless, we reduce the need to screen a large number of clones using small-scale expression, and can focus on characterizing only the most promising clones with more time-consuming qualitative methods such as flow cytometry and fluorescence microscopy.

In our limited trials, we screened around 75 clones for HaTRPA1, and identified only one highly fluorescing clone. However, this might not be a huge problem as several of the less intensely fluorescing clones performed better in the small-scale screen. Still, a larger initial screen than the 50–100 clones previously suggested for membrane proteins [18], would be recommended and the plate screen method is suitable as a high-throughput method.

### Comparing the constructs

The main point of the Δ1-708 HaTRPA1 construct is to enable higher yields of protein, and therefore it is interesting to compare it to HaTRPA1. It is immediately evident that there is a difference in localization between the two constructs when the fluorescent microscopy images are compared (Fig. 4). Other membrane proteins have been reported to localize both to the plasma membrane and in internal structures [22–25] or specifically to accumulate in stacked membranes and perinuclear membranes [26]. It has been proposed that a preferentially internal localization would correlate with protein size and/or the number of transmembrane helices [22]. Since, there is a twofold difference is size between HaTRPA1 and Δ1-708 HaTRPA1, whereas the transmembrane domain is identical in the two constructs, our result would be consistent with the idea that large protein size alone could be sufficient for an internal localization. It is plausible that the difference in localization for our constructs is due to some problem with e.g. folding, or aggregation for HaTRPA1, but we have no direct method of measuring this. Instead, we have to infer any such interpretation from our other findings.

The first observation that we want to highlight is the difference in viability between the Δ1-708 HaTRPA1 and HaTRPA1, both in the small-scale and large-scale fermentations. The percentage of intact cells is lower in Δ1-708 HaTRPA1 cultures, which implies that the protein in some way affects the survival of the expression host. As noted in Fig. 6, the cells are viable at first and give a fluorescent signal when the expression starts, and only later do some of these cells start to have reduced viability. Noteworthy is that the Δ1-708 HaTRPA1 BW30, a clone without GFP, suffers from a similar compromised viability. An interpretation that is close at hand is that the heterologously expressed ion channel is leaky and disturbs the electrochemical gradient over the cell membrane, thereby compromising e.g. the driving force for transport and maintenance of homeostasis in the cytosol. The HaTRPA1 cultures do not suffer from this large decrease in cell viability. This is possibly because the protein is not present in the cell membrane in an open conformation, either due to aggregation, degradation, or its correct targeting to the plasma membrane being disrupted [27]. In *E. coli* it has been reported that membrane proteins with a C-terminal GFP tag that form inclusion bodies rather than folding correctly, end up without any fluorescence from the GFP tag [28]. If this is applicable in *P. pastoris*, the high GFP fluorescence would argue against any major misfolding in either HaTRPA1 or Δ1-708 HaTRPA1.

If we, instead of viability, compare the levels of GFP signal in HaTRPA1 and Δ1-708 HaTRPA1, we get an inconsistent answer. In the small-scale expression, HaTRPA1 has a higher level, whereas in the fed-batch expression, Δ1-708 HaTRPA1 has the higher level. This could be linked to the more controlled environment in the bioreactor, like the pH, higher oxygen level and continuous addition of methanol, providing more optimal conditions for growth and less stress to Δ1-708 HaTRPA1 cells already sensitive due to detrimental effects of the expressed protein. In line with this, the fraction of damaged cells is higher in the small-scale induction in 50 mL tubes compared to induction in the bioreactor. Considering the GFP signal and the fraction of intact cell, an early harvest might avoid degradation of the expressed construct if proteases are released within or from damaged cells. Especially Δ1-708 HaTRPA1 would probably benefit of such an adjustment, whereas HaTRPA1 might reach higher functional yields in shake flask culture, as has been reported for an unusual case before [29].

### Purification

Even if the expression levels are sufficient, the most important question is still whether or not intact protein can be extracted from the cells in high enough quantities for structural characterizations. To illuminate this question, our full purification protocol was performed on cells from both constructs. When preparing crude membranes of both constructs, we already see several fluorescing bands around 30 kDa (Fig. 9), which is close in size to free GFP (28 kDa). The fact that there are several smaller bands of variable size slightly larger than GFP, suggests that we have degradation of the protein at this timepoint that is not limited to the GFP tag or TEV site, but rather affects the actual protein of interest. HaTRPA1 with the GFP tag intact, would be expected to show up around 160 kDa, and there are indeed several weak bands, some of which could match that size. However, one should keep in mind that membrane proteins in particular are known to migrate unpredictably and also as multimers on SDS-PAGE [9, 30], and we cannot for sure tell exactly which band corresponds to our protein of interest. We must also keep in mind that if the protein is partially degraded, any multimers will give rise to more than a single band. Δ1-708 HaTRPA1 with the GFP tag intact, on the other hand, would be expected to show up around 82 kDa. There is a clear band on both the Coomassie stained gel, and the fluorescent gel around 60 kDa, which stands out. It is smaller than expected, although as mentioned above, this might be explained by unpredictable migration. Still, the possibility that degradation is a problem here as well must be considered.

In the IMAC step, the mid-range bands for HaTRPA1 and Δ1-708 HaTRPA1 emerge more clearly, as do the lower-sized bands. For HaTRPA1, this is peculiar, as it means that we seem to purify something of comparable size to the Δ1-708 HaTRPA1 band that we focused on before. The smaller bands seem to go from many non-fluorescing bands with weak staining on the Coomassie gel, to fewer bands with stronger staining on the Coomassie gel, most of which do fluoresce, which is consistent with an enrichment by the IMAC. The peak fraction of the SEC is very similar, but with all bands being fainter due to dilution.

Strangely, we do get sharp elution peaks from SEC, but of the exact same size for both HaTRPA1 and Δ1-708 HaTRPA1 (Fig. 8). This is most likely because the HaTRPA1 construct has suffered some type of degradation, not only from losing the C-terminal GFP tag but also other parts, since the nominal size difference between the two constructs is around 75 kDa – much larger than GFP alone. In the peak fraction we still get smaller bands, even though the SEC should theoretically be able to separate those based on size alone. An explanation for this could be that the smaller fragments are held together by none covalent bonds in a folded conformation in the micelle, and only fall apart when denatured in SDS. Whether this degradation is already taking place in the cell, or is mainly occurring during purification and can be prevented by additional protease inhibitors or other adjustments in the purification protocol remains to be investigated.

### Less is more – finding the right solubilization

A related ion channel, TRPV2, also carrying a C-terminal GFP tag, was recently expressed and purified from *P. pastoris*, using a strain deficient in protease A [24]. Interestingly, fluorescing smaller fragments were much more prominent when fos-choline 12 was used in the solubilization compared to other detergents. Thus, it is possible that fos-choline detergents, although successfully used in purifications of functional TRPA1 from man and mosquito [9, 10], expose susceptible regions of some TRPs for proteases in the solubilization step. Hence, milder detergents may be a way forward to avoid degradation of HaTRPA1, even if the yield of the solubilization might suffer. There are also alternatives to detergents, such as the styrene maleic acid (SMA) copolymer that can be used to directly extract membrane proteins together with surrounding lipids from the lipid bilayer to form SMA lipid particles (SMALPs). This method is likely to be more gentle for the protein, but might not be as efficient for solubilization as detergents [31]. Dutta et al. employed a SMA copolymer to isolate two GFP tagged constructs of a plant Na^+^/H^+^ antiporter from *P. pastoris* [32]. Interestingly, both the longer construct and the half the size version with only the transmembrane domain were found to localize intracellularly. The purification was not straight forward, as the binding in IMAC was weak and other proteins like the alcohol oxidase were difficult to avoid. Despite the gentle extraction, smaller GFP tagged fragments were observed, of some were already present in the membrane suspension. Hence, there might not be one solution to the solubilization problem, instead these alternative methods have to be tried out to find the best protocol for each protein.

### Conclusion and future direction

We have used several methods to investigate the expression of HaTRPA1 in *P. pastoris*, and show that the relationship between expression levels in different steps, and the final result is more complicated than expected. On-plate screening is useful as a good way of weeding out lowly expressing clones, but struggles to pinpoint jack-pot clones that perform best in small-scale liquid culture. Similarly, the constructs deviate in how well they perform in fed-batch culture compared to the small-scale liquid culture. Fluorescence microscopy and flow cytometry are underexploited methods in evaluating clones that revealed interesting differences in localization between HaTRPA1 and Δ1-708 HaTRPA1, which might have relevance for folding, maturation and purification of the protein. Especially the possibility to follow the effect of protein expression on cell viability is a promising prospect that can be used to optimize the time point for harvesting a culture. Improvements of the yield and integrity of the expressed HaTRPA1 will pave the way for structural determination and identification of binding sites for agonists, which can be exploited to develop putative anti-feeding agents that may be used alone or in combination with other practices, like mounding and physical barriers to protect conifer seedlings from the pine weevil.

### Electronic supplementary material

Below is the link to the electronic supplementary material.

Supplementary Material 1

## Data Availability

No datasets were generated or analysed during the current study.

## Abbreviations

AITC: Allyl isothiocyanate
ARD: Ankyrin Repeat Domain
CFU: Colony Forming Units
GFP: Green Fluorescent Protein
HaTRPA1: *Hylobius abietis* Transient Receptor Potential Ankyrin 1
PI: Propidium Iodide
