# Supplementary material for: Evaluation of heterologous expression in Pichia pastoris of Pine Weevil TRPA1 by GFP and flow cytometry
Source: Microb Cell Fact. 2024 Apr 12;23:110. doi: 10.1186/s12934-024-02382-5 (PMC11015645; doi:10.1186/s12934-024-02382-5)
Supplement: Supplementary file 1 — Supplementary Material 1 [file 12934_2024_2382_MOESM1_ESM.docx]

# Supplemental data


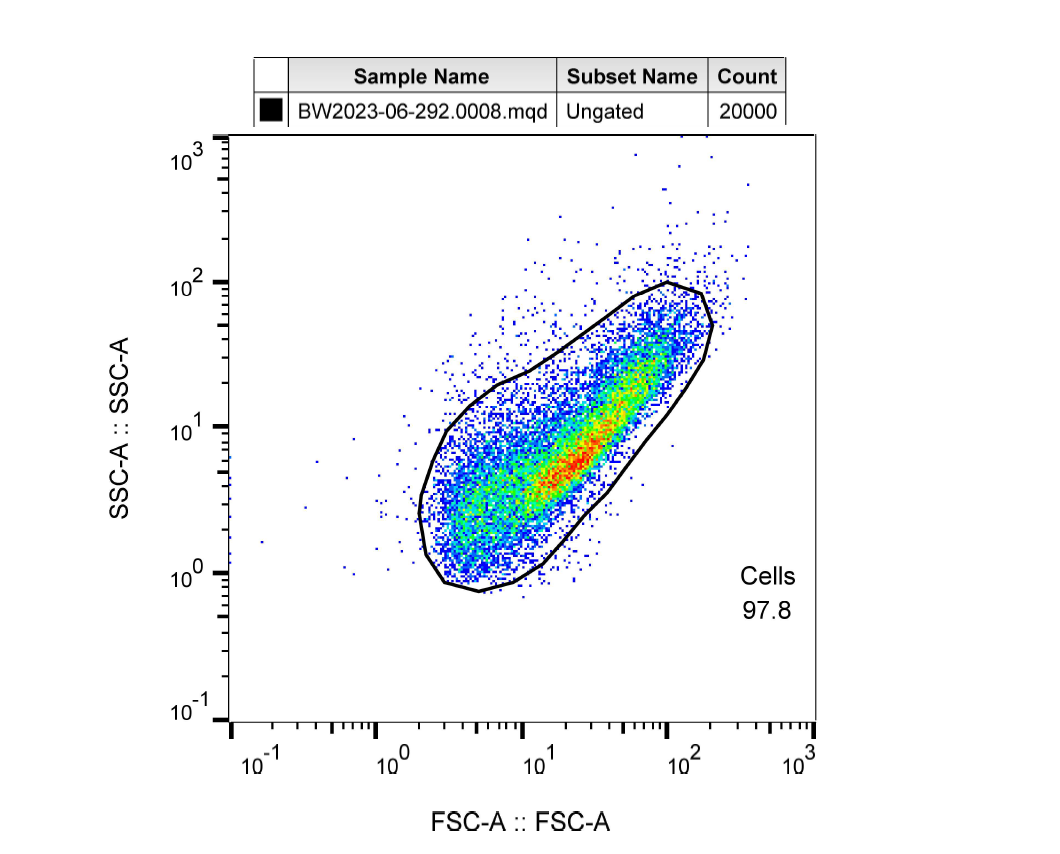


Figure S1. Gating to sort out noise and cell debris by FSC-A and SSC-A. Clone BW30.


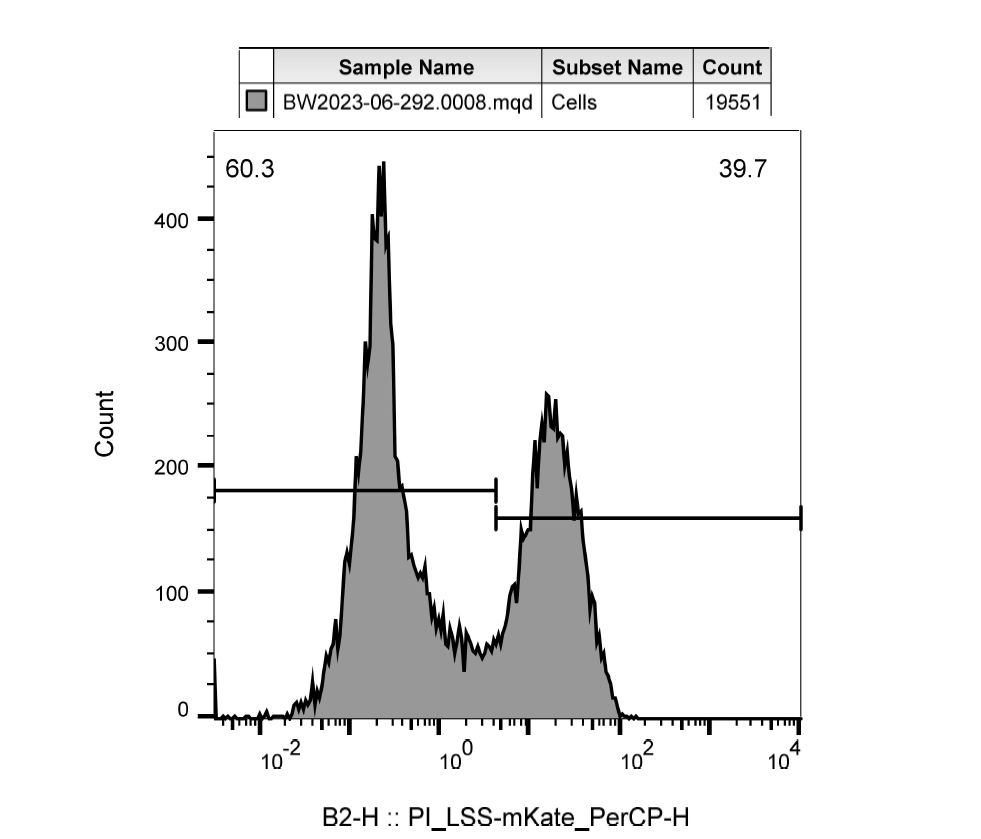


Figure S2. Gating to separate PI positive and negative cells. Clone BW30.

Figure S3. Percentage of intact cells plotted versus GFP signal (not gated on FL3-H) in small scale expression of clones expressing HaTRPA1 (Blue) and Δ1-708 HaTRPA1 (Orange).


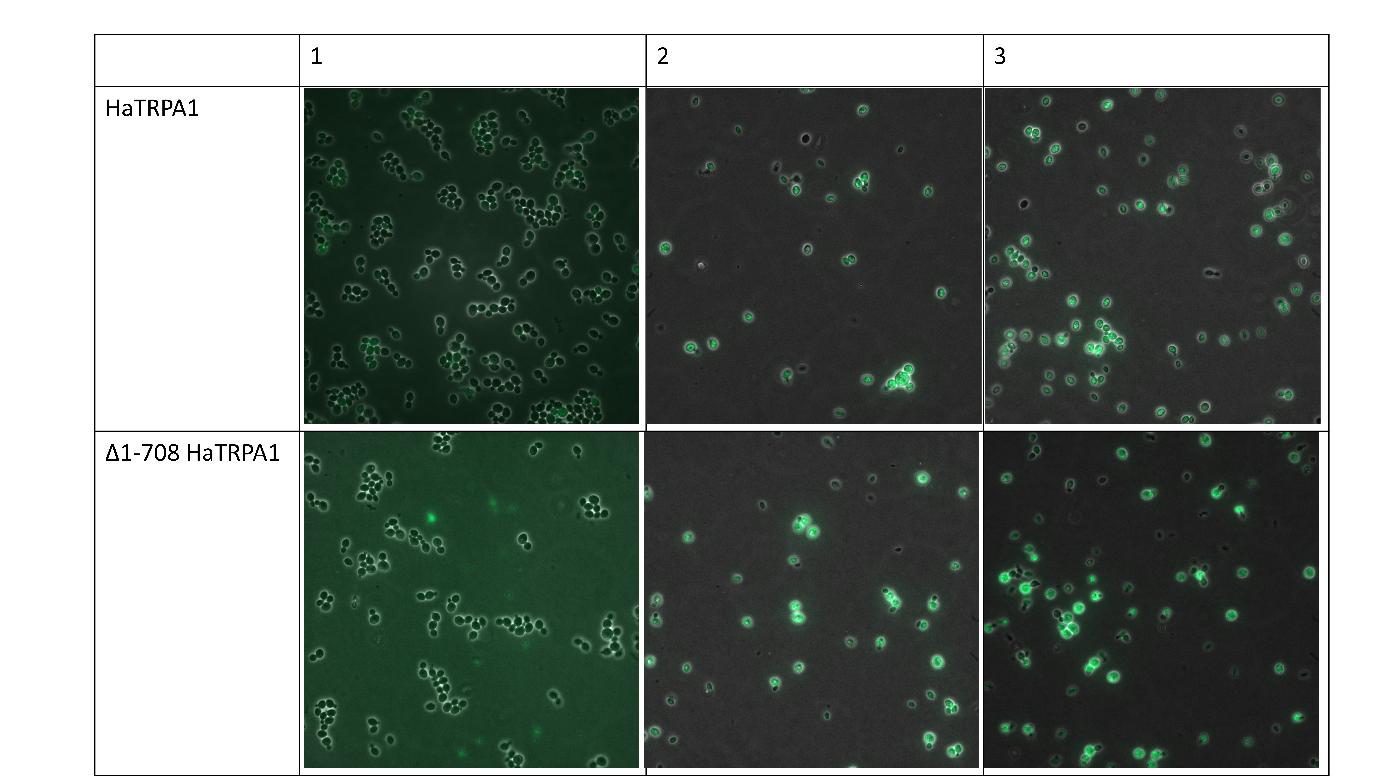


Figure S4. Fed-batch fluorescence microscopy images. Images of samples from the fed-batch culture. (1) 4.8 hours before induction. (2) 19.85 hours after induction. (3) 44.5 hours after induction.
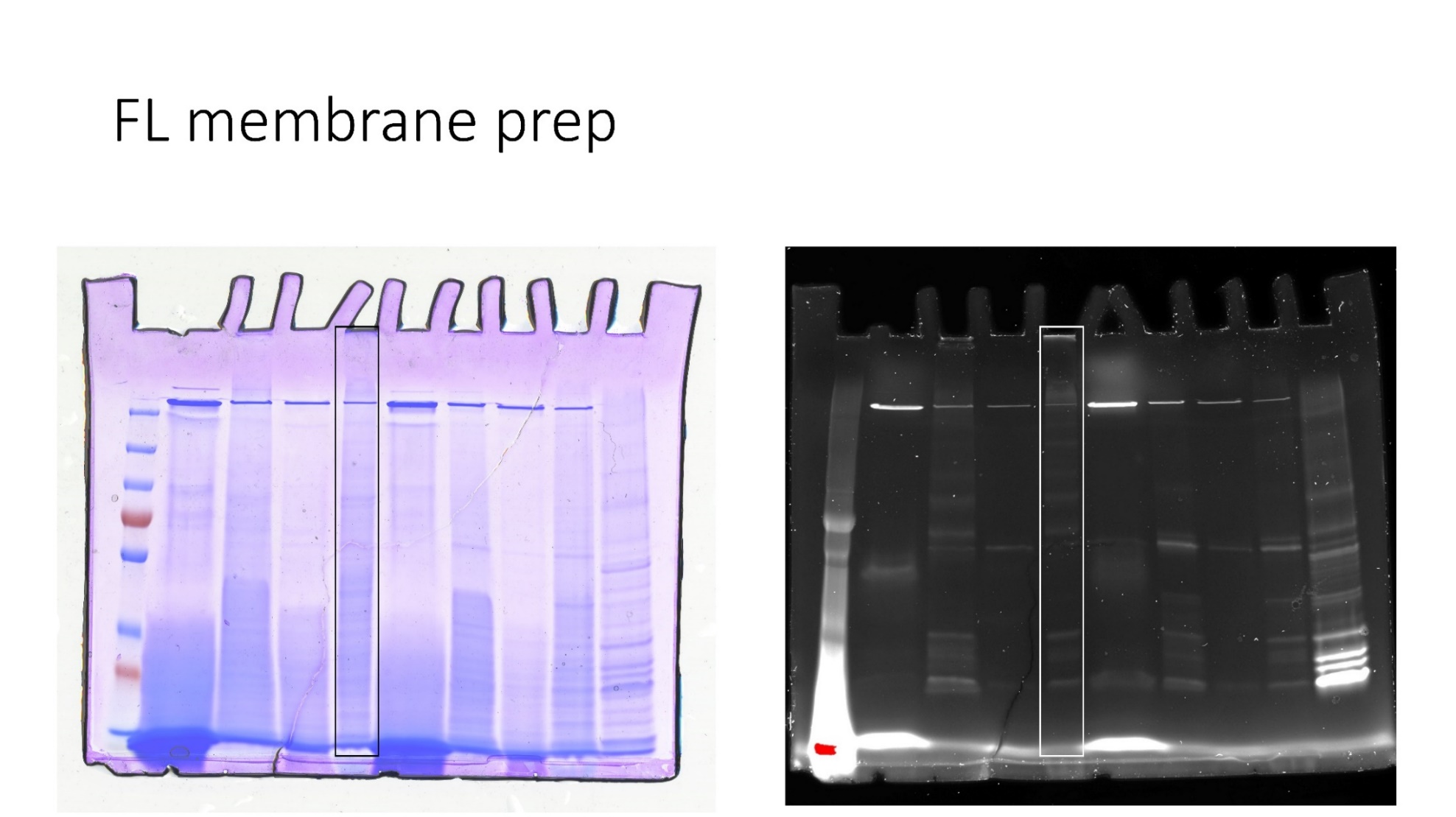

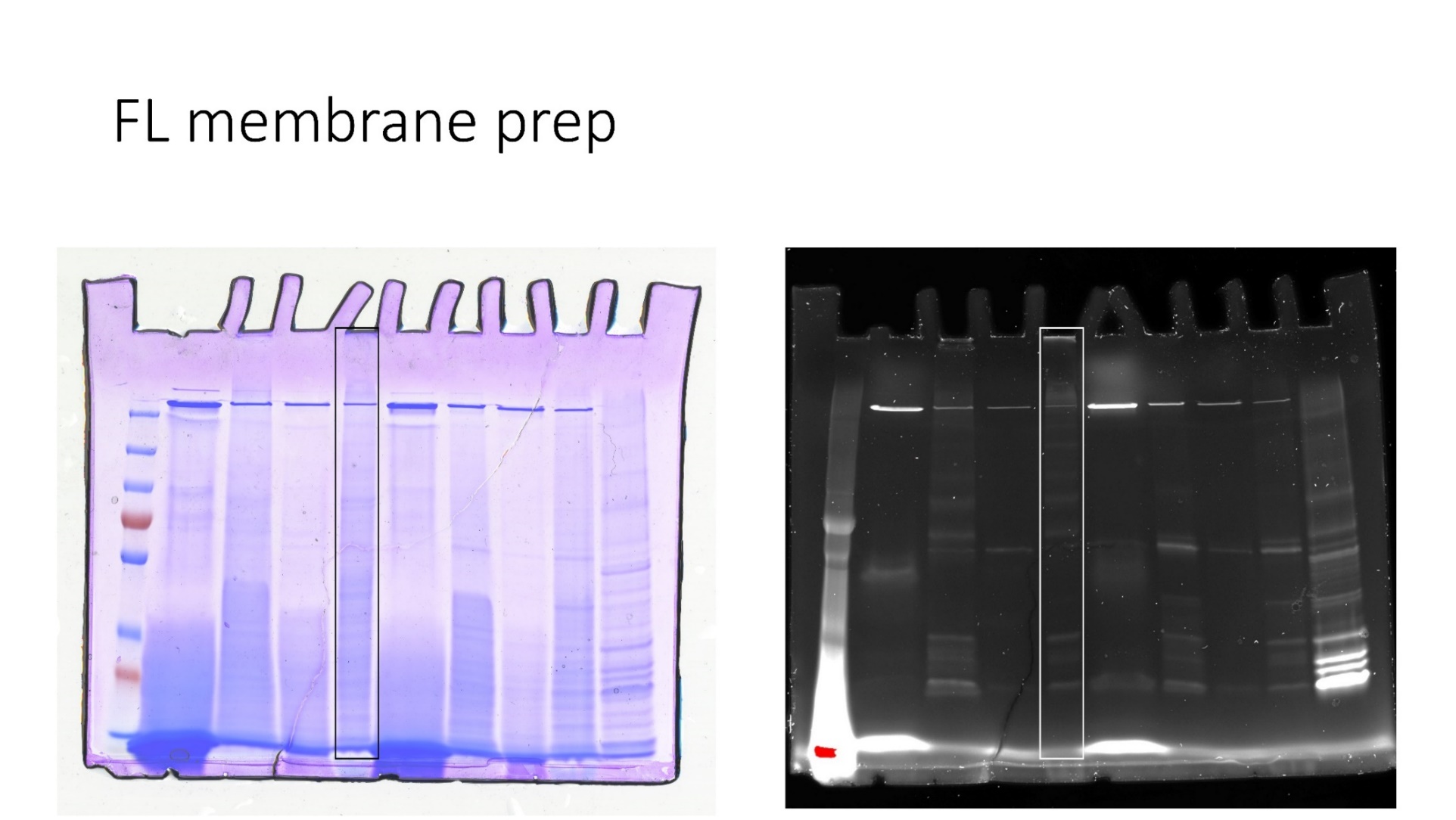


Figure S5. SDS-PAGE, complete gel: HaTRPA1 membrane preparation. Top: Coomassie stained gel. Bottom: fluorescence gel. The lane highlighted by the rectangle is incorporated into Figure 9 lane 2 and 3.


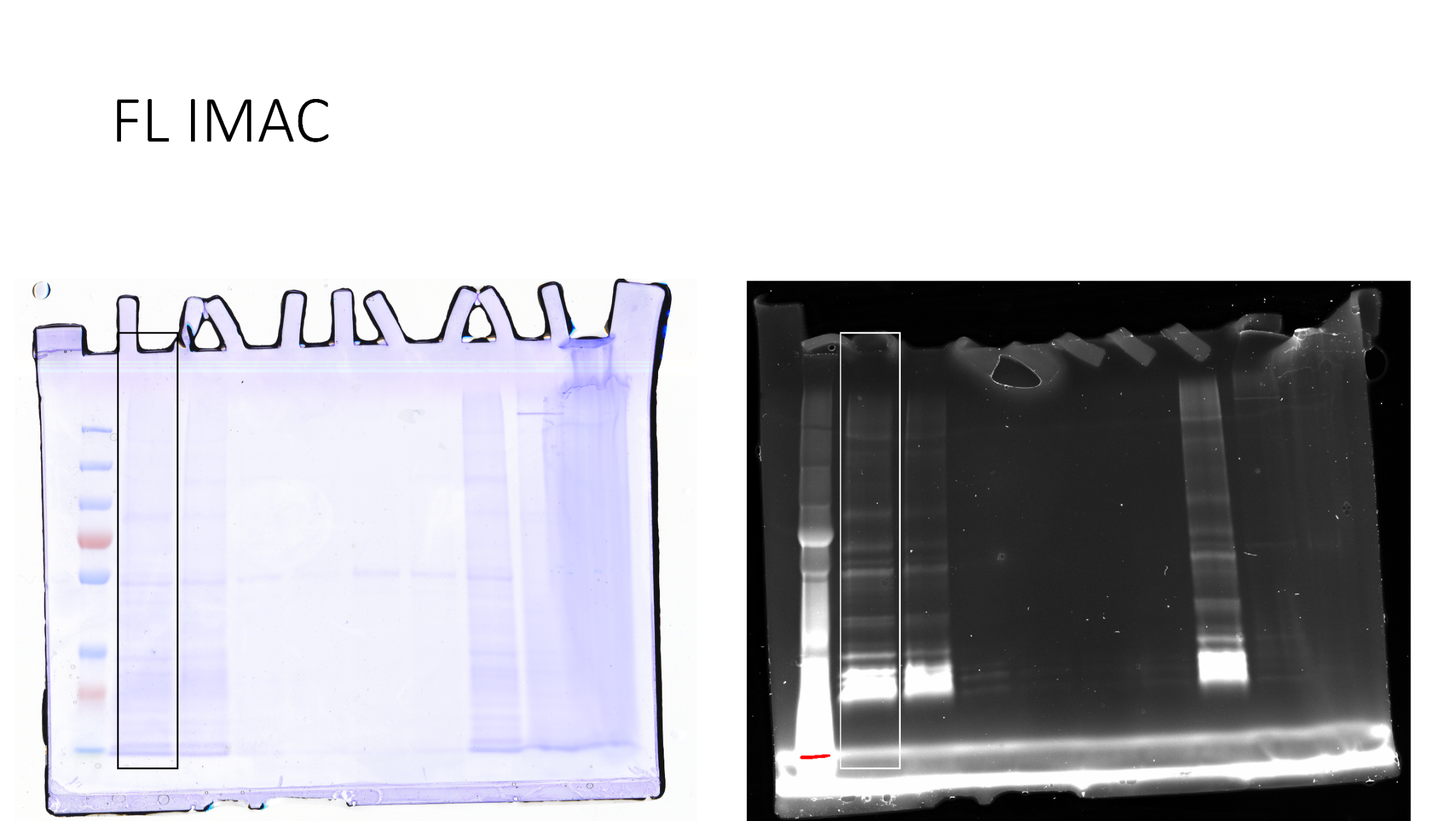


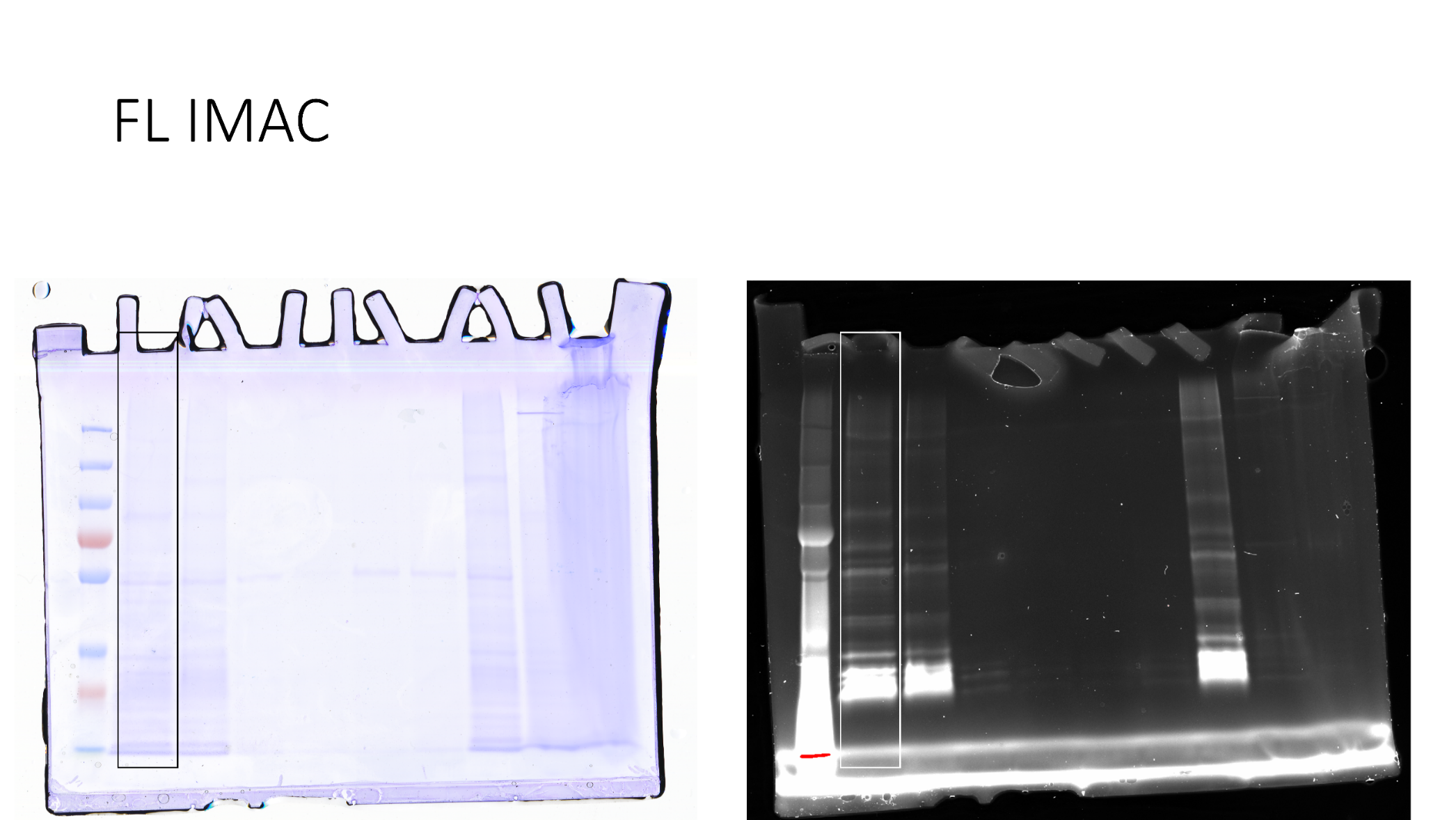


Figure S6. SDS-PAGE, complete gel: HaTRPA1 IMAC. Top: Coomassie stained gel. Bottom: fluorescence gel. The lane highlighted by the rectangle is incorporated into Figure 9 lane 4 and 5.


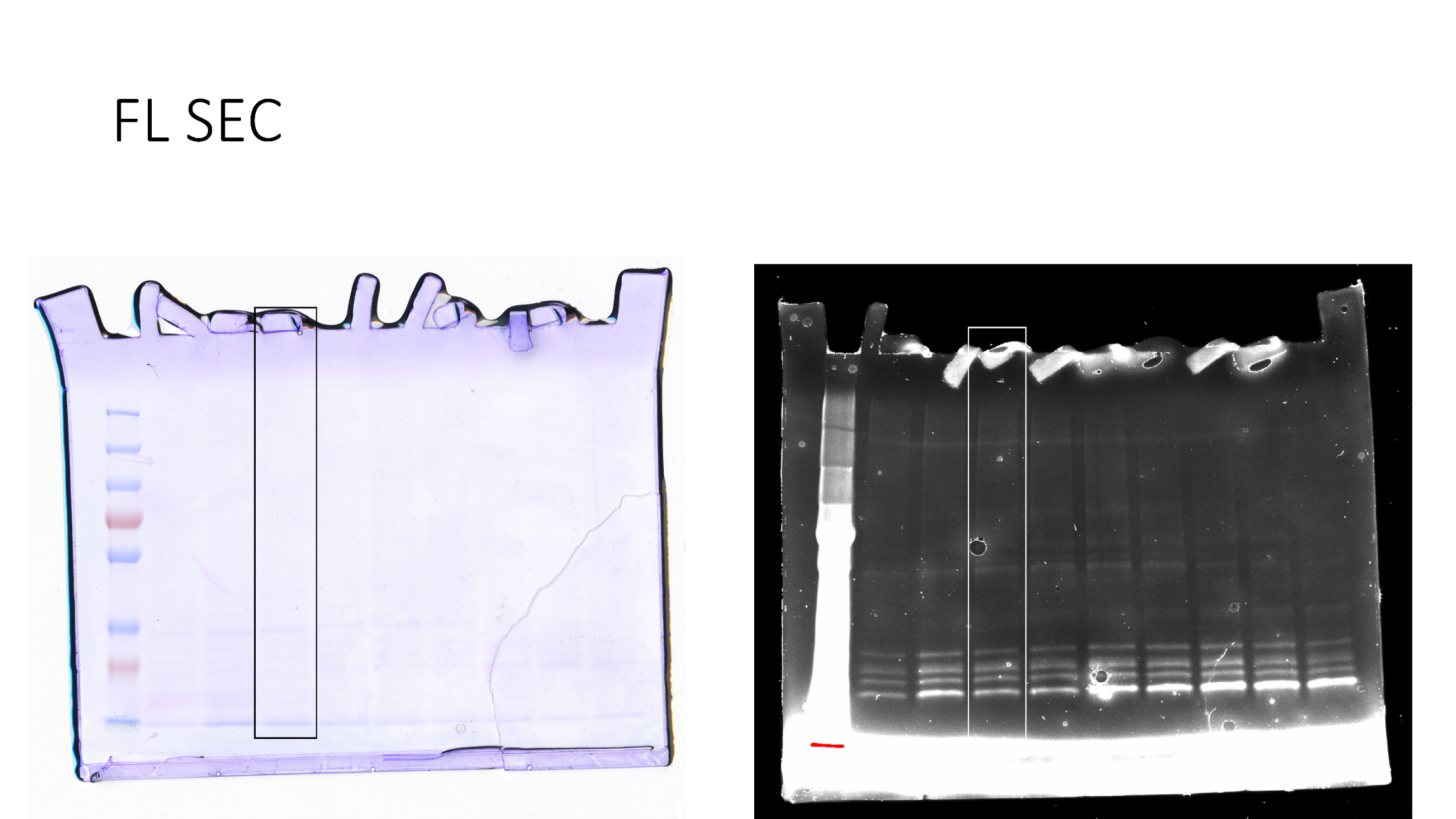


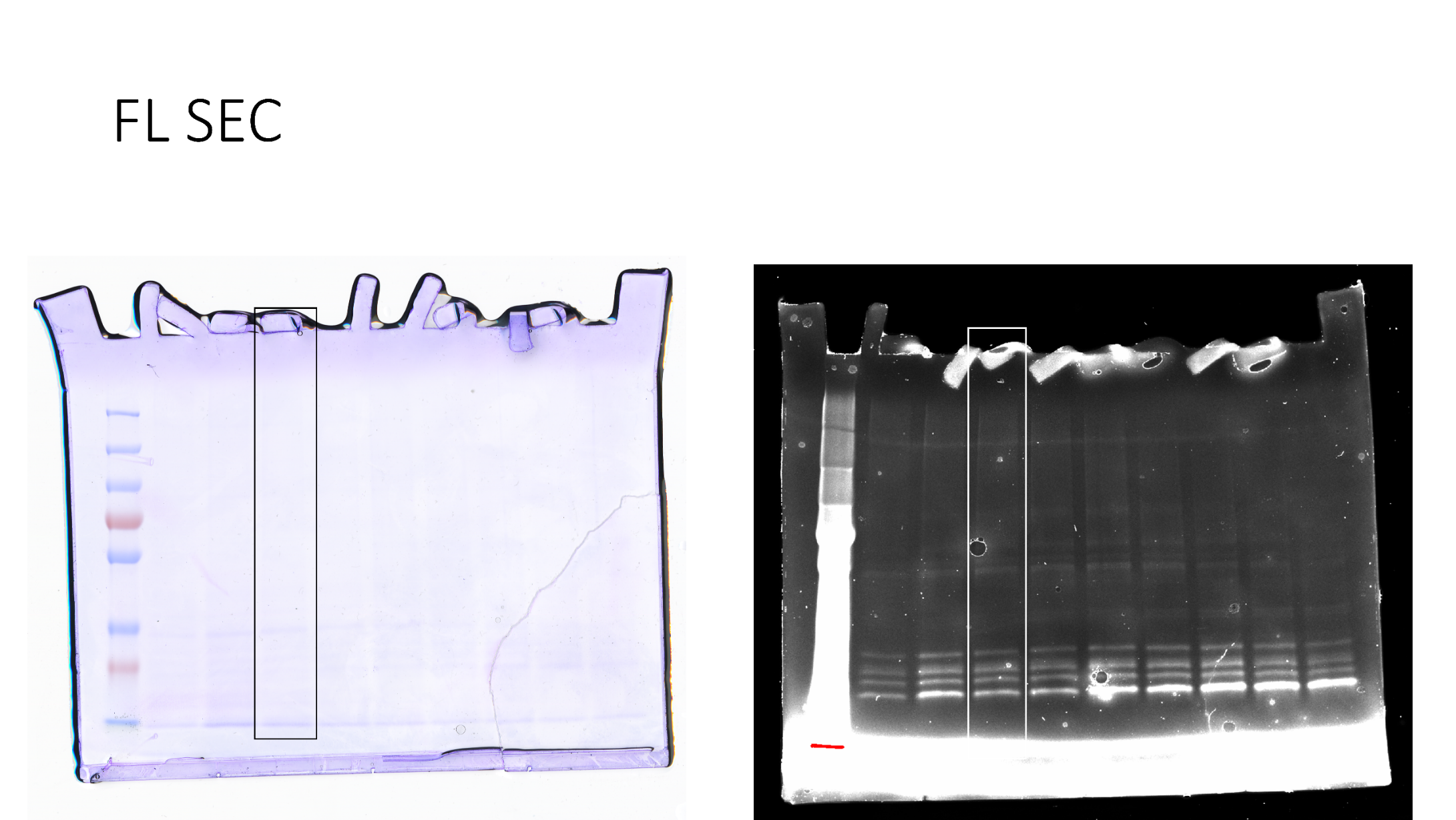


Figure S7. SDS-PAGE, complete gel: HaTRPA1 SEC. Top: Coomassie stained gel. Bottom: fluorescence gel. The lane highlighted by the rectangle is incorporated into Figure 9 lane 6 and 7.


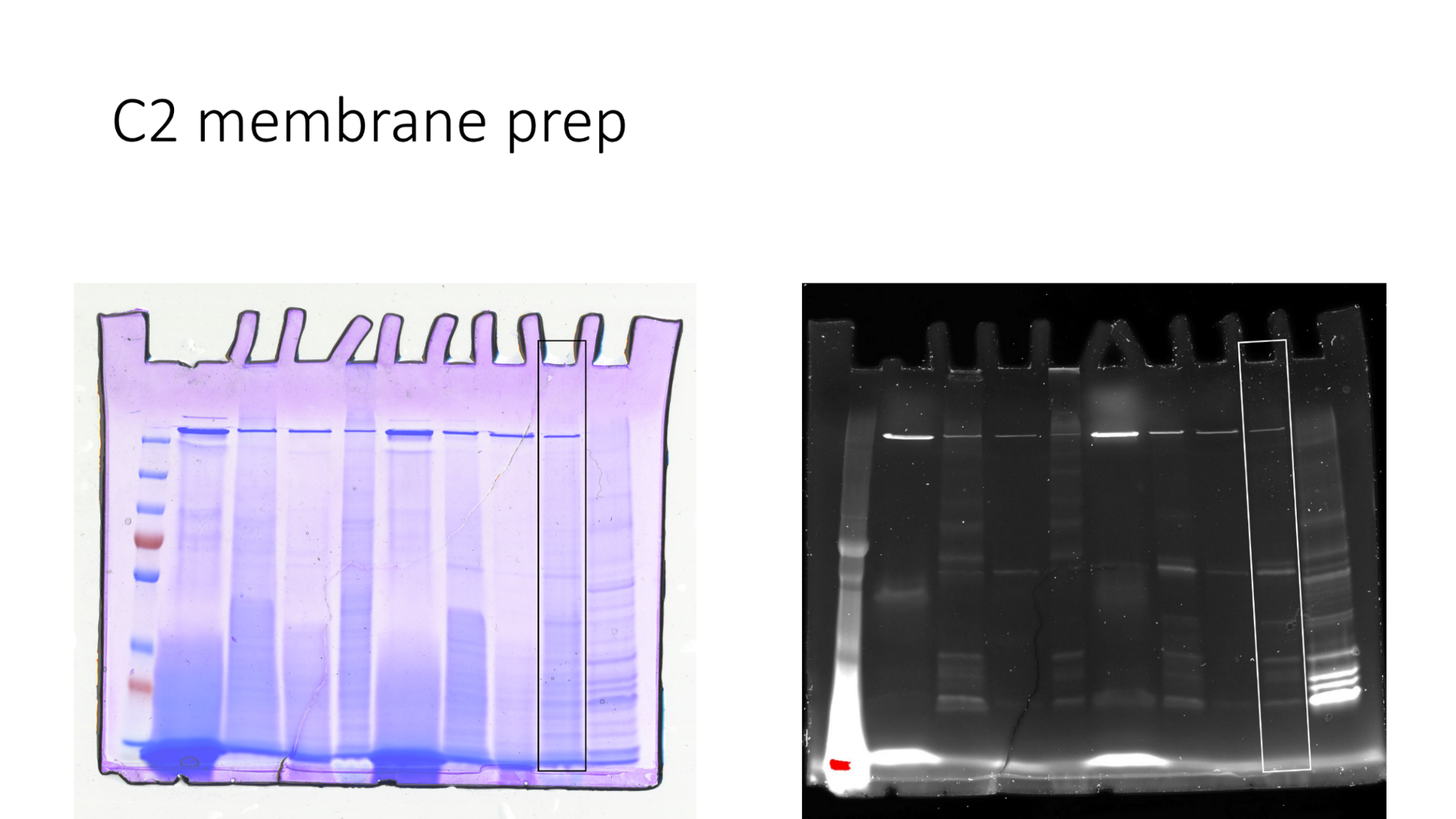


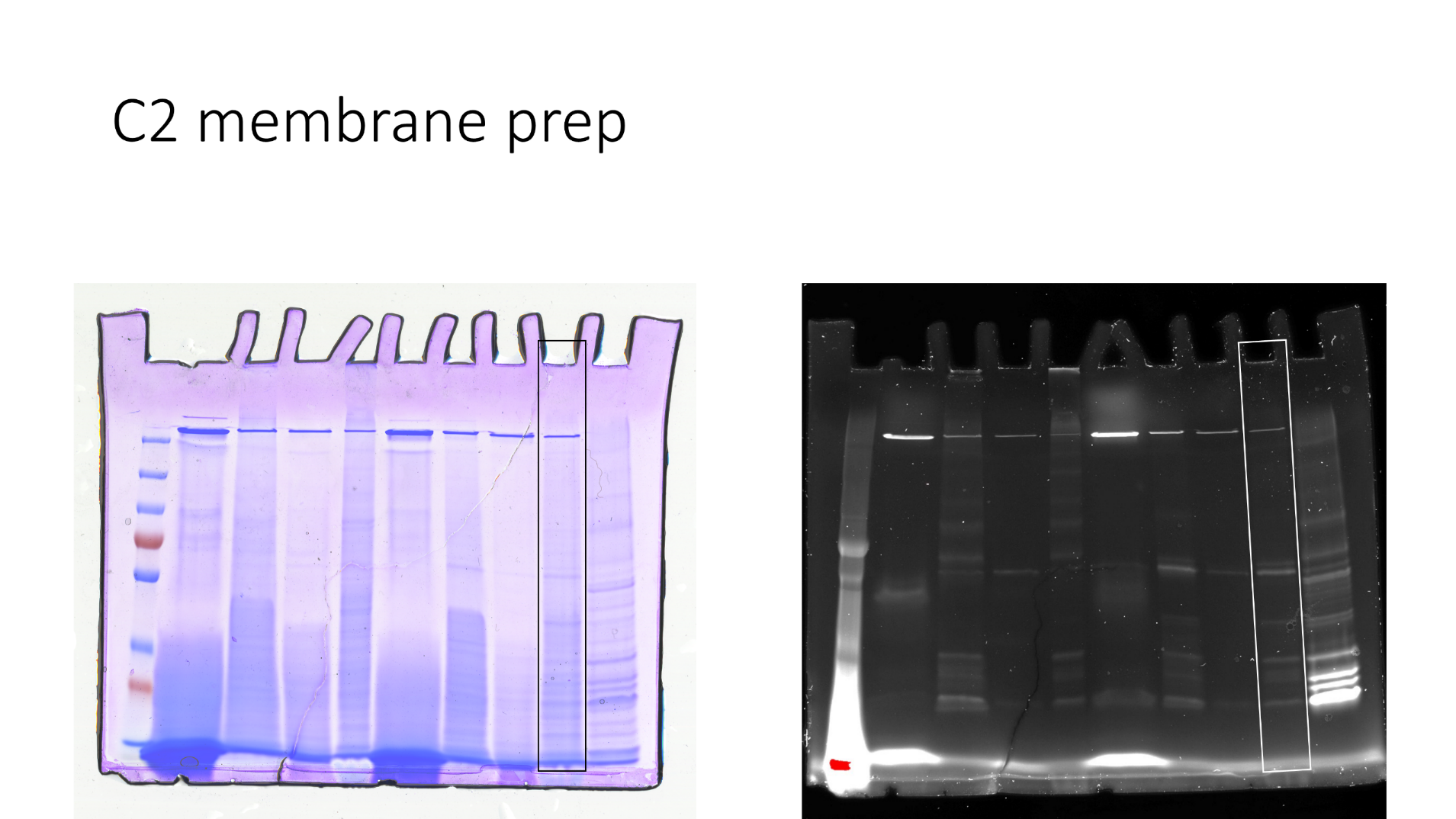


Figure S8. SDS-PAGE, complete gel: Δ1-708 HaTRPA1 membrane preparation. Top: Coomassie stained gel. Bottom: fluorescence gel. The lane highlighted by the rectangle is incorporated into Figure 9 lane 9 and 10.


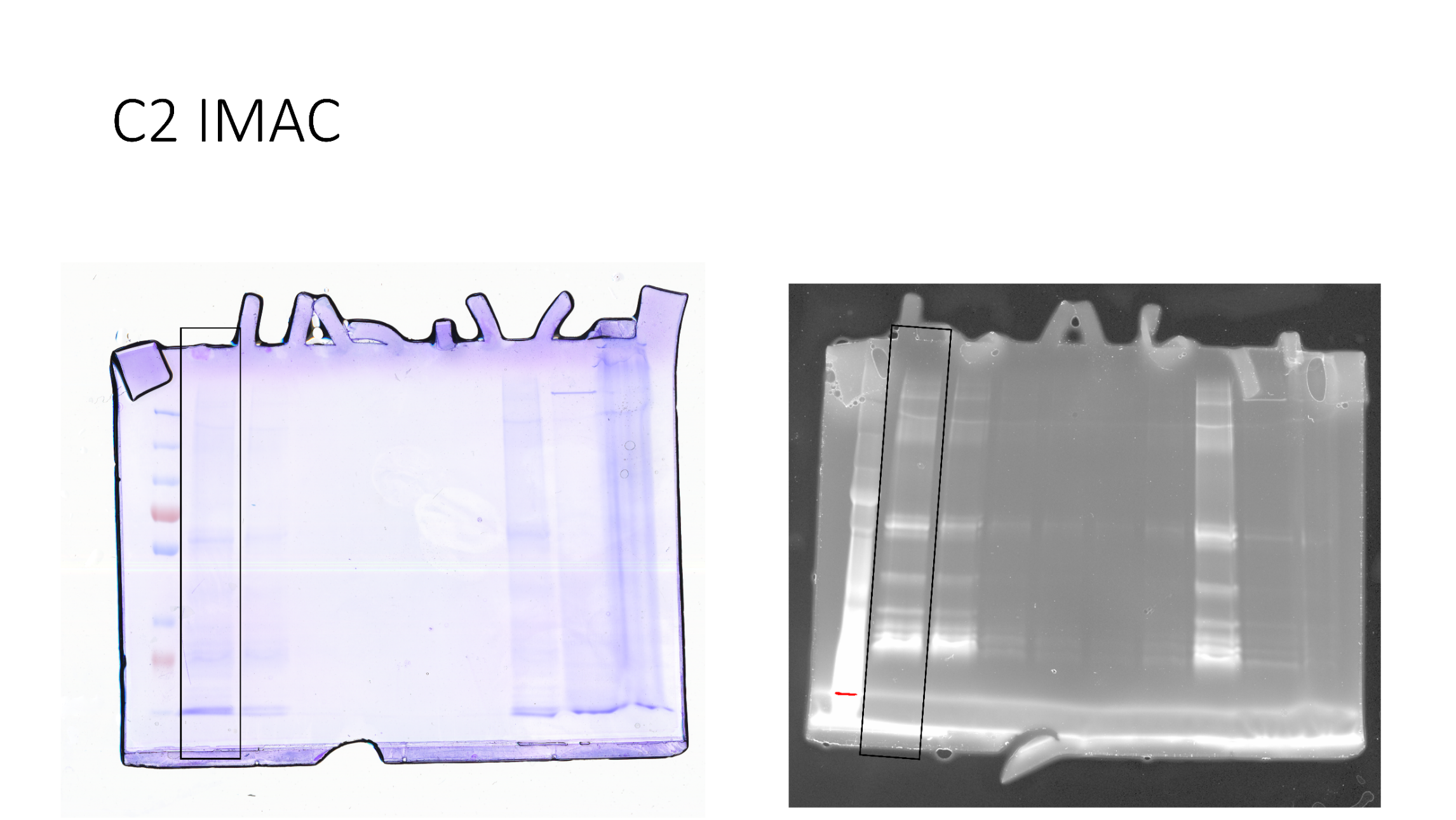


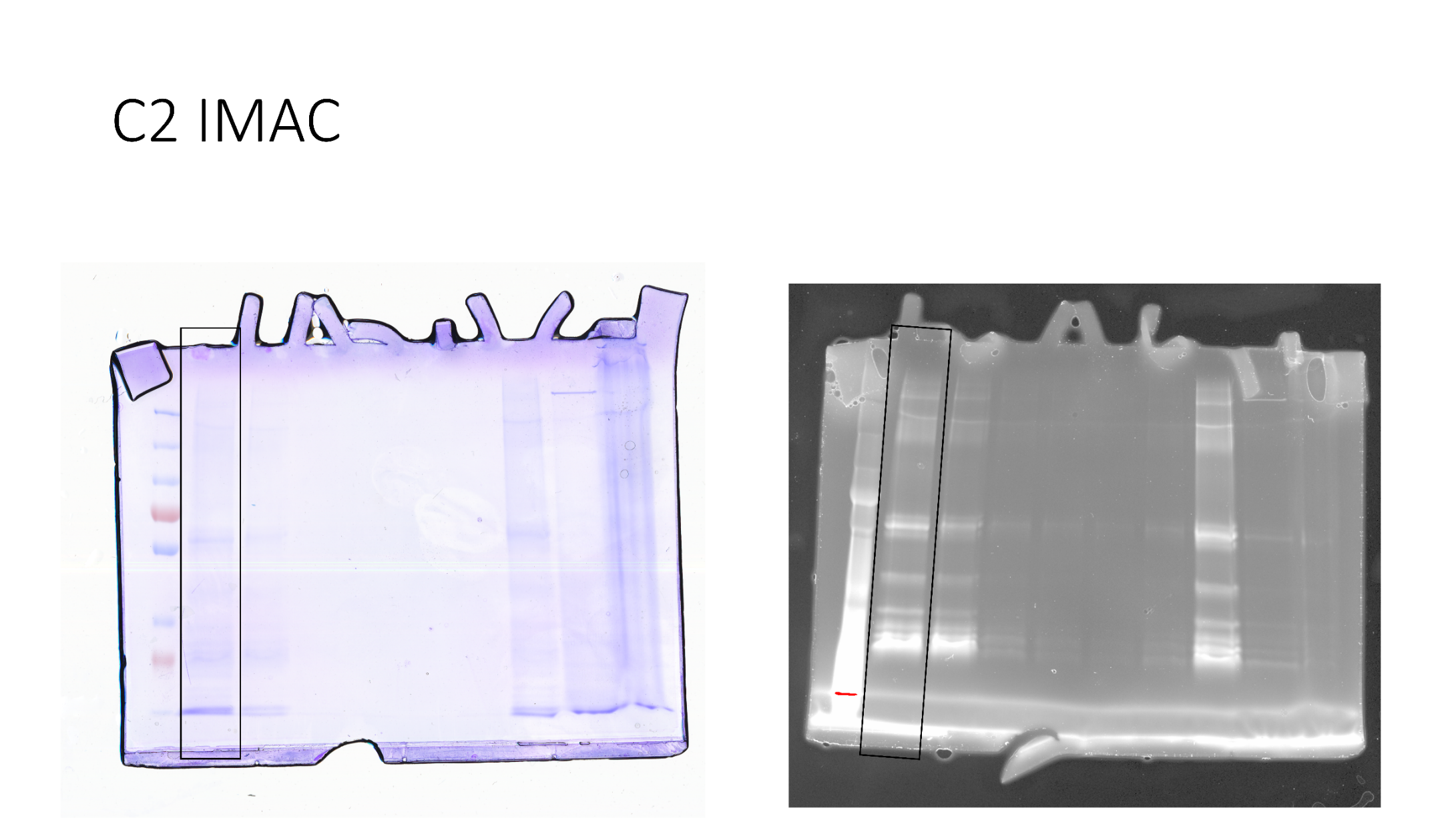


Figure S9. SDS-PAGE, complete gel: Δ1-708 HaTRPA1 IMAC. Top: Coomassie stained gel. Bottom: fluorescence gel. The lane highlighted by the rectangle is incorporated into Figure 9 lane 11 and 12.


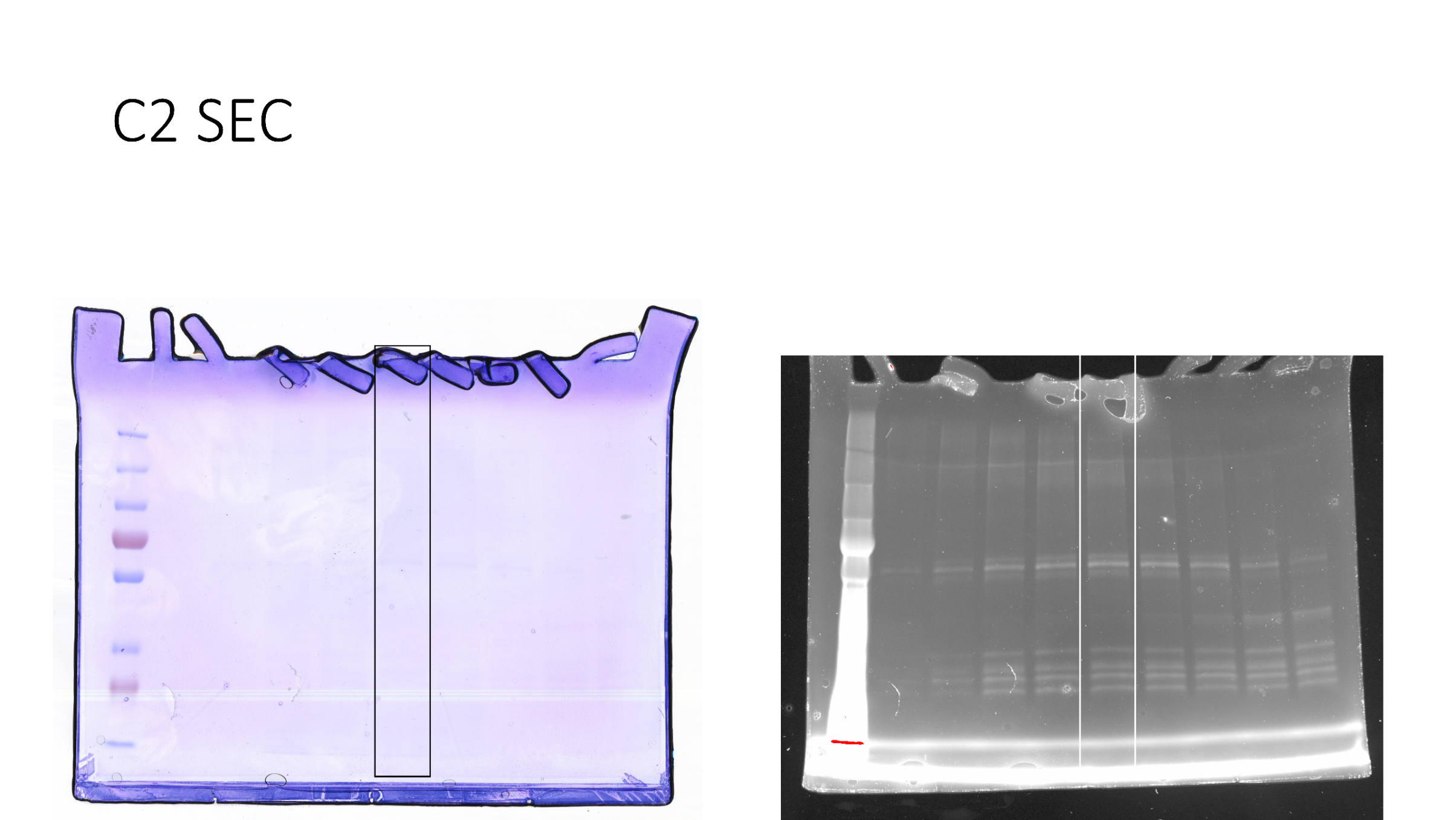


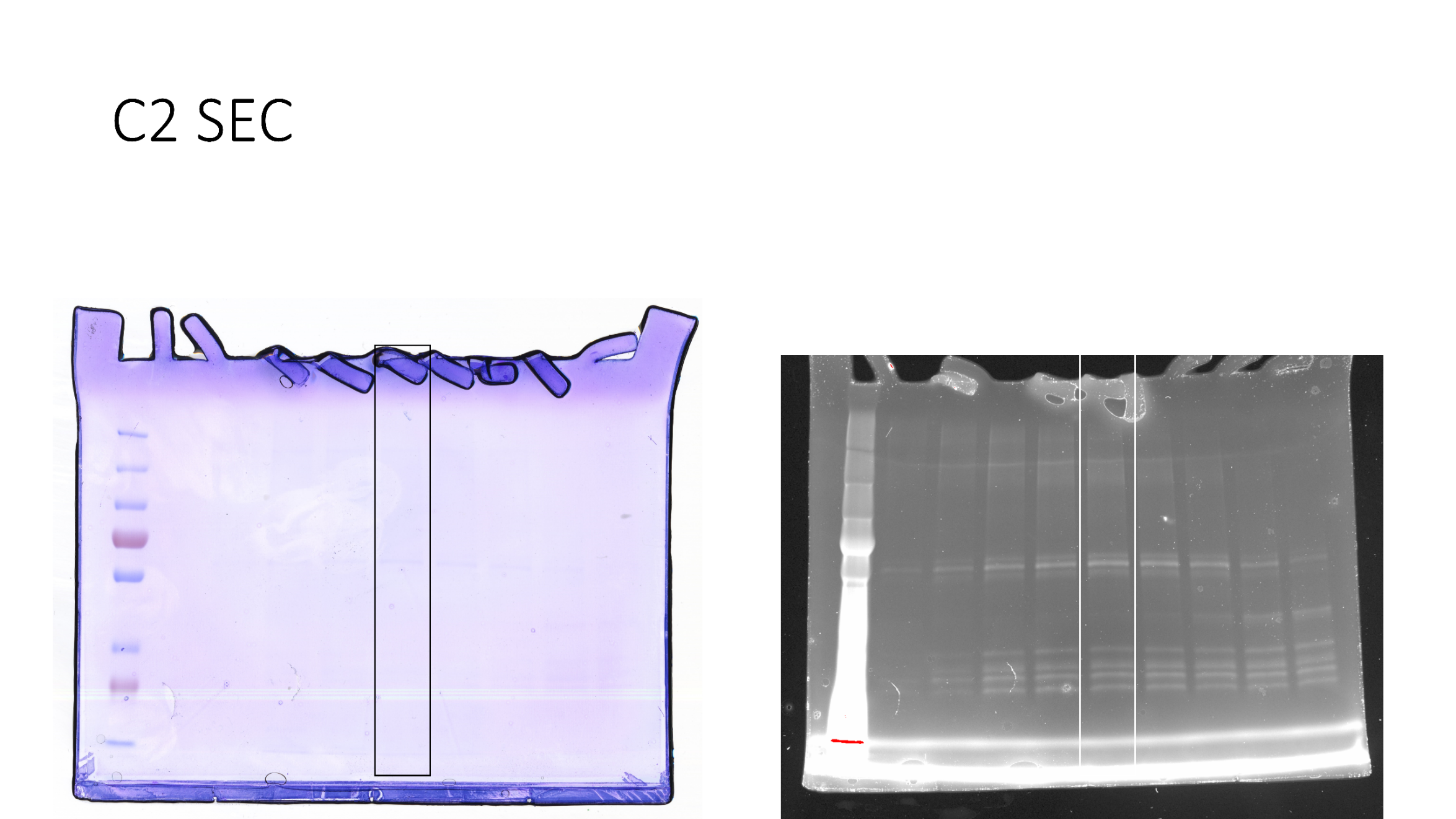


Figure S10. SDS-PAGE, complete gel: Δ1-708 HaTRPA1 SEC. Top: Coomassie stained gel. Bottom: fluorescence gel. The lane highlighted by the rectangle is incorporated into Figure 9 lane 13 and 14.

Table S1. Data from microtiter fluorescence measurements.

| Sample | Raw data (RFU) |
| --- | --- |
| HaTRPA1 supernatant A | 5526 |
| HaTRPA1 supernatant B | 5284 |
| HaTRPA1 supernatant C | 5497 |
| HaTRPA1 pellet A | 43280 |
| HaTRPA1 pellet B | 36410 |
| HaTRPA1 pellet C | 33921 |
| Δ1-708 HaTRPA1 supernatant A | 7721 |
| Δ1-708 HaTRPA1 supernatant B | 6325 |
| Δ1-708 HaTRPA1 supernatant C | 6052 |
| Δ1-708 HaTRPA1 pellet A | 31069 |
| Δ1-708 HaTRPA1 pellet B | 29342 |
| Δ1-708 HaTRPA1 pellet C | 29547 |

Table S2. Data from FCM on small-scale.

| Clone | Mean GFP  (not gated on FL3-H) | Mean GFP  (gated on FL3-H) | PI- (%) |
| --- | --- | --- | --- |
| Δ1-708 BW30 | 1.01 | 0.19 | 60.28847629 |
| Δ1-708 2.1.12 | 1 | 0.25 | 98.78474003 |
| Δ1-708 2.1.35 | 22.9 | 11.3 | 59.24498416 |
| Δ1-708 1.2.1 | 34.4 | 8.46 | 23.61731628 |
| Δ1-708 1.2.2 | 29.9 | 8.55 | 22.83323064 |
| Δ1-708 2.2.37 | 23.8 | 10.9 | 42.22931247 |
| HaTRPA1 2.3.28 | 1 | 0.23 | 98.04745554 |
| HaTRPA1 2.3.12 | 22.6 | 16 | 93.45827985 |
| HaTRPA1 1.2.1 | 54.5 | 38 | 86.49719549 |
| HaTRPA1 1.2.2 | 41.3 | 30.1 | 91.57011175 |
| HaTRPA1 1.2.3 | 34.8 | 22.2 | 89.34987389 |

Supplemental text file 1. Amino acid sequence of constructs with colors indicating different regions. HaTRPA1 (grey), TEV site (yellow), GFP (green), His-tag, spacers and modified start of the protein (white).

HaTRPA1 construct: 1420 residues 160 kDa, cleaved by TEV 1169 aa 131.8 kDa

>FL_HaTRPA1_TEV_GFP_8xHis

MSNSFRSLVSAYGTESELQNMLPSDVENGQDKTSNGEAVCSIASSPYRILRAAESGNLDMFQRLYIQDPSRLSIQDPRGRTTAHQAASRNKINILTFINQQGGDLNAQDNVGNTPLHVAVESEALDAVDYLLTVGVKTDILNEKKQAPVHLATELSKISVLERMAKYKDKIDIEQGGEHGRTALHIAAIYDHDACARVLISDFGASPRKPCNNGYYPIHEAAKNASSKTMEVFLAWGESLGCTRDEMISFYDAEGNVPLHSAVHGGDIRAVELCIRSGAKISTQQHDLSTPVHLACAQGAIEIVKIMFQMQPEEKMACLASCDVQKMTPLHCAAMFDHPEIVEYLISEGADCNPIDKERRSPLLLAALRGGWKTVHSLIRLGADINIKDINKRNVLHLVVMNGGQLEQFAAEAKSKESLLQLLNEKDLTGCSPLHYASREGHIRSLENLIRLGACINLKNNNNESPLHFAARYGRYNTVKQLLDSEKGNFIINESDGEGMTPLHIASQQGHTRVVQLLLNRGALLHRDHNGRNPLHLAAMNGYTQTIELLLSVHSHLLDQLDKDGNTALHLATMENRPNVIAQLLSMKCKLLYNQTEMSAIDYAIYYKYLEAALAMVTHEDRAEEIMVLKSPKNPCVSLALIASMPRVFEAVQDKCIIKANCKKDSKLFSIKYSFCCLQCNTVTEEIDEKTGDKIVEKEPTPLPALNAMVAHGRVELLAHPLSQKYLQMKWNSYGKYFHLTNLLFYCIFLGSITCFSSQLMEHEKALNFSYIKYSNMTTEQKYAERKSEILDVQINYSMYICAMAILVFIVLNGAREIAQMIQQKCMYFFNPINLVTWCLYGATIVMVLPIFGGEIYEIQFSFASLVVFLSWFNLLLLLQRFDQVGIYVVMFLEILQTLIKVLMVFSILIIAFGLAFYILLSRGDHLSFKTIPMALIRTFSMMLGEIDFLGTYVKPYYLSNKEENTFLPFPIPAFLILGLFMVLMPILLMNLLIGLAVGDIESVRRNAQLKRLAMQVVLHTELERKLPRRWLERVDKAEITEYPNESKCKKGILDFILKKWFGSPFSEDSDIVMENSEDYVVSELAKTKNKLRQISQALETQNQFLRLIVQKMEIKTEADDIDEGVPLRNTSHGHSSKWTSPKIRKKIKSVLSFSNRANSTLEENLYFQSAGGSVSKGEELFTGVVPILVELDGDVNGHKFSVSGEGEGDATYGKLTLKFICTTGKLPVPWPTLVTTLTYGVQCFSRYPDHMKQHDFFKSAMPEGYVQERTIFFKDDGNYKTRAEVKFEGDTLVNRIELKGIDFKEDGNILGHKLEYNYNSHNVYIMADKQKNGIKVNFKIRHNIEDGSVQLADHYQQNTPIGDGPVLLPDNHYLSTQSALSKDPNEKRDHMVLLEFVTAAGITLGMDELYKHHHHHHHH

Δ1-708 HaTRPA1 construct (C2 =Δ1-708): 712 residues, 81.8 kDa, cut by TEV 461 aa, 53.5 kDa

>C2_HaTRPA1_TEV_GFP_8xHis

MSAHGRVELLAHPLSQKYLQMKWNSYGKYFHLTNLLFYCIFLGSITCFSSQLMEHEKALNFSYIKYSNMTTEQKYAERKSEILDVQINYSMYICAMAILVFIVLNGAREIAQMIQQKCMYFFNPINLVTWCLYGATIVMVLPIFGGEIYEIQFSFASLVVFLSWFNLLLLLQRFDQVGIYVVMFLEILQTLIKVLMVFSILIIAFGLAFYILLSRGDHLSFKTIPMALIRTFSMMLGEIDFLGTYVKPYYLSNKEENTFLPFPIPAFLILGLFMVLMPILLMNLLIGLAVGDIESVRRNAQLKRLAMQVVLHTELERKLPRRWLERVDKAEITEYPNESKCKKGILDFILKKWFGSPFSEDSDIVMENSEDYVVSELAKTKNKLRQISQALETQNQFLRLIVQKMEIKTEADDIDEGVPLRNTSHGHSSKWTSPKIRKKIKSVLSFSNRANSTLEENLYFQSAGGSVSKGEELFTGVVPILVELDGDVNGHKFSVSGEGEGDATYGKLTLKFICTTGKLPVPWPTLVTTLTYGVQCFSRYPDHMKQHDFFKSAMPEGYVQERTIFFKDDGNYKTRAEVKFEGDTLVNRIELKGIDFKEDGNILGHKLEYNYNSHNVYIMADKQKNGIKVNFKIRHNIEDGSVQLADHYQQNTPIGDGPVLLPDNHYLSTQSALSKDPNEKRDHMVLLEFVTAAGITLGMDELYKHHHHHHHH

Construct in the BW30 clone similar to Δ1-708 HaTRPA1 but without GFP-tag: 480 residues, 55.7 kDa

>C2_10xHis_TEV_HaTRPA1

MSHHHHHHHHHHDSNGIPTENLYFQGAMVAHGRVELLAHPLSQKYLQMKWNSYGKYFHLTNLLFYCIFLGSITCFSSQLMEHEKALNFSYIKYSNMTTEQKYAERKSEILDVQINYSMYICAMAILVFIVLNGAREIAQMIQQKCMYFFNPINLVTWCLYGATIVMVLPIFGGEIYEIQFSFASLVVFLSWFNLLLLLQRFDQVGIYVVMFLEILQTLIKVLMVFSILIIAFGLAFYILLSRGDHLSFKTIPMALIRTFSMMLGEIDFLGTYVKPYYLSNKEENTFLPFPIPAFLILGLFMVLMPILLMNLLIGLAVGDIESVRRNAQLKRLAMQVVLHTELERKLPRRWLERVDKAEITEYPNESKCKKGILDFILKKWFGSPFSEDSDIVMENSEDYVVSELAKTKNKLRQISQALETQNQFLRLIVQKMEIKTEADDIDEGVPLRNTSHGHSSKWTSPKIRKKIKSVLSFSNRANST
